# Computational design of an ultrapotent deltacoronavirus miniprotein inhibitor

**DOI:** 10.1073/pnas.2533456123

**Published:** 2026-04-29

**Authors:** Nathan G. Avery, Courtney N. Yoshiyama, Ashley L. Taylor, Young-Jun Park, Daniel Asarnow, Lisa Perruzza, Jack T. Brown, Davide Corti, Fabio Benigni, Tyler N. Starr, David Veesler

**Affiliations:** ^a^Department of Biochemistry, University of Washington, Seattle, WA 98195; ^b^Department of Biochemistry, University of Utah School of Medicine, Salt Lake City, UT 84112; ^c^HHMI, University of Washington, Seattle, WA 98195; ^d^Humabs Biomed SA, a Subsidiary of Vir. Biotechnology, Bellinzona 6500, Switzerland

**Keywords:** coronaviruses, minibinders, protein design, spike glycoprotein, PDCoV

## Abstract

Multiple spillovers of porcine deltacoronavirus (PDCoV) into humans in Haiti highlight its zoonotic potential and the need for targeted interventions. No approved vaccines or therapeutics are available for use in humans against any DCoVs. Here, we report the de novo design of PDCoV miniprotein inhibitors (aka minibinders, MBs) and show that one of them, MB11, binds with picomolar affinity to the PDCoV receptor-binding domain (RBD). MB11 potently inhibits PDCoV, outcompeting monoclonal antibodies, and cross-reacts with and broadly neutralizes a panel of distantly related DCoVs. We determined a cryoelectron microscopy structure of MB11 bound to the PDCoV RBD which reveals the molecular basis of broad DCoV neutralization through interference with host receptor engagement. Deep mutational scanning of the PDCoV RBD reveals that MB11 has a high barrier to viral escape with only few mutations mediating escape without dampening APN receptor binding. MB11 resists stringent biochemical stresses, including high temperature, low pH, and proteolysis, which may enable delivery to various tissues for viral inhibition. This work delineates a prime candidate for clinical evaluation against PDCoV infection and for pandemic preparedness.

## Significance

Multiple porcine deltacoronavirus (PDCoV) spillovers occurred in Haiti and there are currently no vaccines or therapeutics approved for use in humans. We computationally designed PDCoV miniprotein inhibitors and identified one (MB11) that potently and broadly neutralizes distantly related delta-coronaviruses. MB11 is resistant to multiple biochemical stresses, an ideal property for easy storage and delivery. These data pave the way for developing therapeutics to prepare for possible future PDCoV outbreaks.

Coronaviruses (CoVs) are viruses in the coronaviridae family that can cause moderate to severe enteric, respiratory, hepatic, and neurologic disease in a wide range of animals and are classified into five genera: alpha, beta, gamma, delta, and epsilon (1–4). Delta-CoV (DCoVs) are part of a recently described genus (3) for which birds are thought to be the natural host reservoir (2). CoVs recurrently spill over from natural host reservoirs to humans with the most recent major outbreaks including the SARS (5–8) and MERS epidemics (9) and the COVID-19 pandemic, all caused by beta-CoVs (10, 11). Moreover, several CoVs are now endemic in humans (12) and cause numerous respiratory infections (13): alpha-CoVs NL63 (14) and 229E (15) and beta-CoVs HKU1 (16) and OC43 (17).

CoVs utilize the spike (S) glycoprotein to enter host cells and initiate infection. The S protein is composed of two functional subunits termed S_1_ and S_2_. The S_1_ subunit is responsible for host cell receptor binding (18–24) and the S_2_ subunit promotes fusion of the viral and host membranes (25, 26). Since the S protein is crucial for promoting cell entry, alteration of receptor recognition allows to modulate host and tissue tropism and can lead to spillover (27–29). Targeting the S protein can block CoVs at the earliest stage of the viral life cycle and prior research has identified that inhibition of entry by vaccine-elicited antibodies or monoclonal antibodies can provide protection against CoVs (30–33).

Porcine delta coronavirus (PDCoV) was discovered in Hong Kong in 2012 (2) and has since caused several outbreaks in pigs around the world (34). PDCoV uses the aminopeptidase N (APN) receptor for cell entry and has spilled over into humans in Haiti through multiple zoonotic transmission events (35, 36). The PDCoV S architecture and the molecular basis of APN engagement have been defined (24, 37, 38), providing a blueprint to design countermeasures targeting PDCoV. A small number of PDCoV monoclonal neutralizing antibodies with limited breadth and small molecule therapeutics with moderate potency have been described (39–44). This work showed that PDCoV S-directed antibodies that interfere with host receptor engagement have the most potent neutralizing activity (39–41). Although these studies provided proof-of-principle for S-directed viral inhibition, potent and broad inhibitors of viral entry are needed for pandemic preparedness in case of future DCoV zoonotic spillovers. Currently, there are no approved vaccines or therapeutics for use in humans against any DCoV.

De novo designed miniproteins or minibinders (MBs) have emerged as promising inhibitors with potent in vitro neutralizing activity and in vivo protective efficacy in mice against SARS-CoV-2 (45, 46) and MERS-CoV (47). Here, we report the de novo design of MBs targeting the PDCoV RBD and show that one of them, MB11, potently inhibits PDCoV and several distantly related DCoVs, outperforming known neutralizing antibodies. MB11 imposes a high barrier for emergence of escape mutants and is resistant to multiple biochemical stresses, which are ideal properties for large-scale deployment. Cryoelectron microscopy (cryo-EM) analysis of MB11 bound to the PDCoV RBD validated the designed target and revealed the mechanism of PDCoV neutralization through interference with receptor engagement. These data suggest that MB11 is a promising preclinical PDCoV inhibitor that could be stockpiled for pandemic preparedness.

## Results

### Computational Design of PDCoV Miniprotein Inhibitors.

**Fig. 1.**
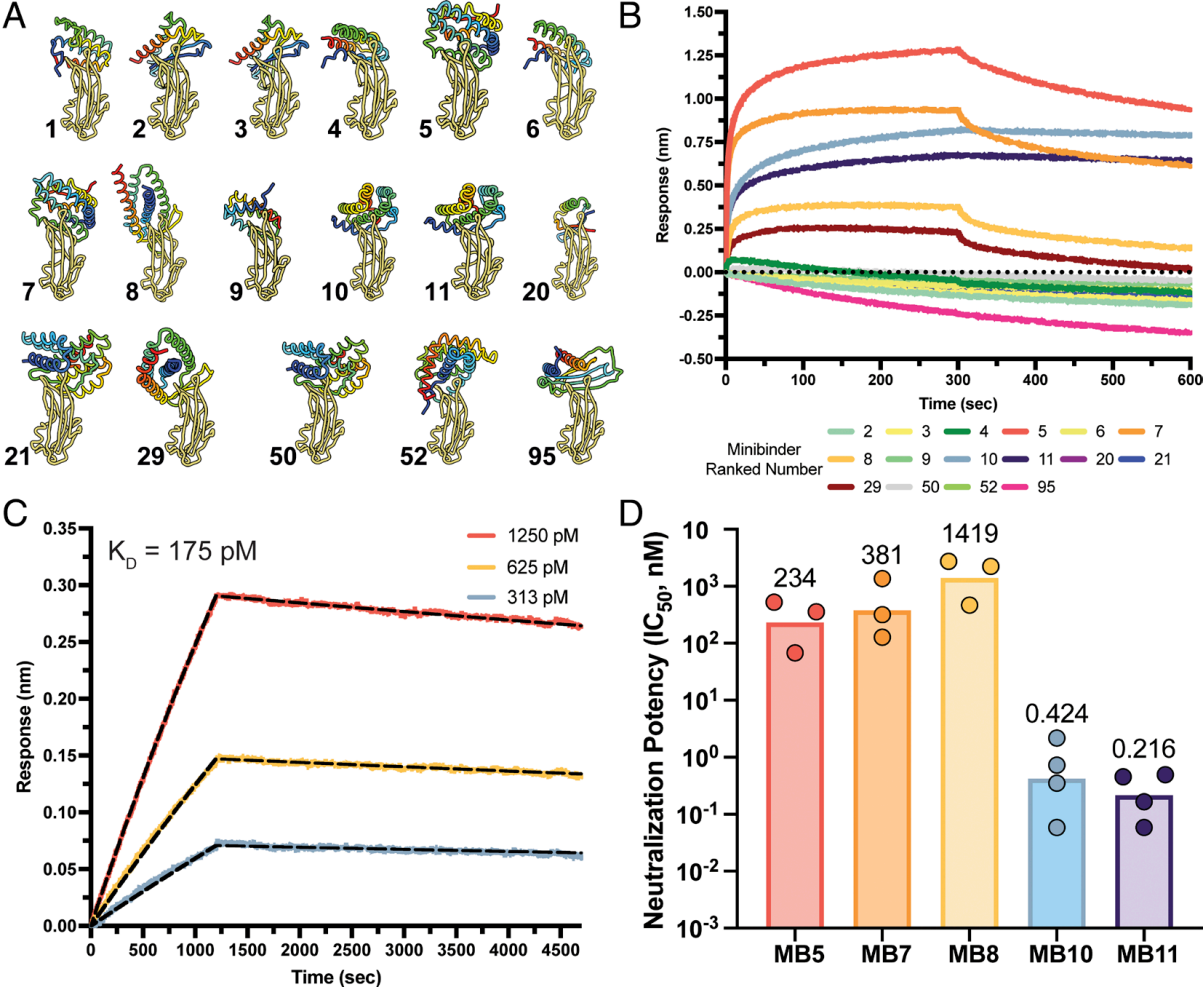
Computational design of PDCoV miniprotein inhibitors. (*A*) Ribbon rendering of the predicted structures of the PDCoV_IL121_2014_ RBD (gold) in complex with the MBs selected for experimental testing (rainbow-colored from N- to C-terminus). The index next to each structure indicates the minibinder name. (*B*) BLI screen for binding of PDCoV_IL121_2014_ S_1_-Fc at a concentration of 500 nM to MBs immobilized at the surface of HIS1K biosensors. One batch of MBs and PDCoV S_1_-Fc were used for the screening experiment. (*C*) BLI analysis of binding kinetics of the PDCoV_IL121_2014_ RBD at multiple concentrations to the MB11 immobilized at the surface of streptavidin biosensors. The PDCoV RBD concentrations used are indicated with the color key and the fit to the data using a 1:1 binding model with global fit is shown as dashed black lines. K_D_: equilibrium dissociation constant. Independent batches of MB11 and PDCoV RBD were used for two biological replicates. (*D*) Neutralizing activity expressed as half-maximal inhibitory concentration (IC_50_) of each MB determined using PDCoV_IL121_2014_ S VSV pseudoviruses and HEK293T target cells transiently expressing galline APN. Each data point represents a biological replicate obtained with distinct batches of minibinders and of pseudoviruses. The bar represents the geometric mean of 3 to 4 biological replicates.

To design inhibitors of PDCoV, we used Bindcraft (48) to generate de novo MBs ranging from 54 to 190 amino acid residues and targeting the RBD receptor-binding loops of the PDCoV_IL121_2014_ RBD. A total of 101 designs passed the Bindcraft filters and we subsequently predicted their structures in complex with the PDCoV_IL121_2014_ RBD using AlphaFold3 (49). We ranked the designs based on predicted alignment error, interface predicted template modeling (iPTM), and visual inspection of the contacts formed at the predicted interface between each MB and the RBD. Out of the seventeen MBs selected for recombinant expression in *Escherichia coli* (*SI Appendix*, Table S1), sixteen expressed at high levels and were used to screen for binding to the PDCoV_IL121_2014_ S_1_ subunit fused to human Fc (S_1_-Fc) using biolayer interferometry (BLI, Fig. 1 *A* and *B*). We identified six MBs binding to PDCoV_IL121_2014_ S_1_ and two of them (MB10 and MB11) stood out due to their slow offrates (Fig. 1*B*). Given that MB10 and MB11 were selected from the same AF2 hallucination design trajectory (i.e. they share the same predicted interface with the RBD), we selected MB11 for further characterization due to its greater monodispersity relative to MB10 (*SI Appendix*, Fig. S1 *A and B*). BLI analysis of binding of the PDCoV_IL121_2014_ RBD to immobilized MB11 yielded an equilibrium dissociation constant (K_D_) of 155 ± 28 pM and a half-life of 16 ± 12 h (mean ± SD) (Fig. 1*C* and *SI Appendix*, Fig. S2 and Table S2). All MBs for which we detected binding by BLI were further screened for neutralization of VSV pseudotyped with PDCoV_IL121_2014_ S entry into HEK293T cells transiently transfected with galline APN (gAPN). Five MBs (MB5, MB7, MB8, MB10, and MB11) inhibited PDCoV_IL121_2014_ S in a dose-dependent manner with MB10 and MB11 having the most potent neutralizing activity characterized by half-maximal inhibitory concentrations (IC_50_) of 424 and 216 pM, respectively (Fig. 1*D* and *SI Appendix*, Figs. S3 and S4*A*).

### MB11 Broadly Neutralizes Distantly Related DCoVs.

**Fig. 2.**
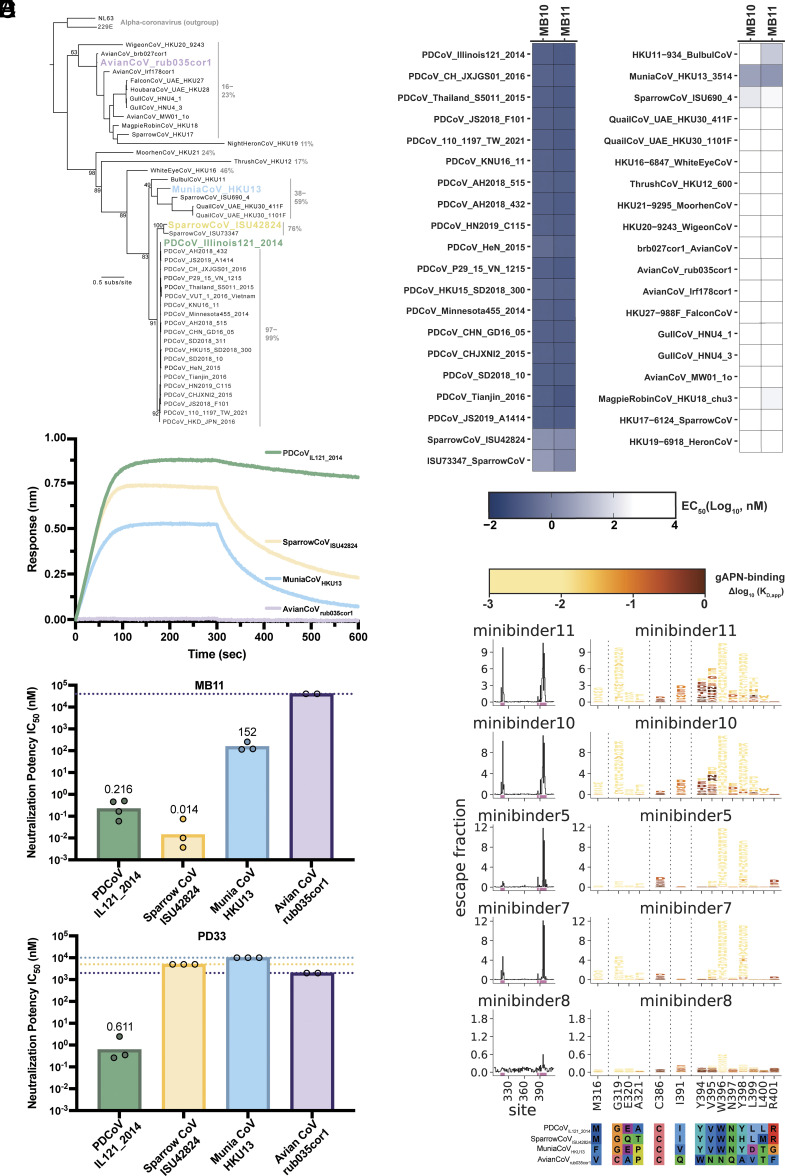
MB11 mediates potent and broad DCoV neutralization. (*A*) Inferred phylogeny of the DCoV genus from RBD amino acid sequence, adapted from (39). The range of sequence identity to PDCoV_IL121_2014_ in each viral clade is indicated. Sequence identity was calculated using Sequence Manipulation Suite: Ident and Sim from bioinformatics.org. (*B*) Heat map showing the breadth of MB10 and MB11 binding to DCoV RBDs expressed on the surface of yeast. (*C*) BLI binding analysis of a panel of DCoV RBDs at a concentration of 50 nM to MB11 immobilized at the surface of streptavidin biosensors. (*D* and *E*) Neutralizing activity expressed as half-maximal inhibitory concentration (IC_50_) of MB11 (*D*) and PD33 Fab (*E*) determined using PDCoV_IL121_2014_, SparrowCoV_ISU42824_, MuniaCoV_HKU13_, and AvianCoV_rub035cor1_ S VSV pseudoviruses and HEK293T cells transiently expressing galline (PDCoV_IL121_2014_), sparrow (SparrowCoV_ISU42824_), or munia (MuniaCoV_HKU13_ and AvianCoV_rub035cor1_) APN orthologs. Dotted lines show the limits of detection of the assays. (*F*) PDCoV_IL121_2014_ RBD escape maps from MB binding obtained by deep mutational scanning. (*Left*) Line plots showing the sum of escape from MB binding due to mutations at each site in the PDCoV_IL121_2014_ RBD. Sites with strong escape are highlighted with pink bars. (*Right*) Logoplots showing individual mutations at each site in the PDCoV_IL121_2014_ RBD that escape with a 10-fold or greater reduction in MB binding. RBD mutations are colored by their orthogonally measured effects on gAPN receptor-binding avidity (increasing light yellow indicates increasingly deleterious and brown indicates neutral or advantageous effects on receptor binding). An alignment of DCoV S sequence isolates used in this manuscript at sites illustrated in the logoplots is shown below.

The marked diversity of the RBD amino acid sequences within the DCoV genus is a grand challenge for the design of countermeasures with broadly neutralizing activity (Fig. 2*A*). To assess the binding breadth of MB10 and MB11, we evaluated cross-reactivity with a panel of yeast surface-displayed RBDs spanning the DCoV phylogenetic diversity. We detected binding to a broad range of RBDs spanning three phylogenetic clades, including the MuniaCoV_HKU13_ RBD, which shares only 59% sequence identity with PDCoV_IL121_2014_ (Fig. 2 *A* and *B* and *SI Appendix*, Fig. S5*A*). Moreover, we observed via BLI that MB11 bound to the PDCoV_IL121_2014_, SparrowCoV_ISU42824_, and MuniaCoV_HKU13_ RBDs but not to the AvianCoV_rub035cor1_ RBD, the latter RBD sharing only 23% sequence identity with PDCoV_IL121_2014_ (Fig. 2*C* and *SI Appendix*, Fig. S6). BLI analysis of SparrowCoV_ISU42824_, and MuniaCoV_HKU13_ RBDs binding to immobilized MB11 yielded K_D_ values of 3.60 nM (*SI Appendix*, Fig. S7 *A–D*) and 13.4 nM respectively (*SI Appendix*, Fig. S7 *E–H*).

Subsequent evaluation of neutralization breadth showed that MB11 inhibited VSV pseudotyped with SparrowCoV_ISU42824_ S and MuniaCoV_HKU13_ S with IC_50_ values of 14 pM and 152 nM, respectively. No neutralization of AvianCoV_rub035cor1_ S VSV was detected with up to 40 µM MB11, concurring with the binding data (Fig. 2*D* and *SI Appendix*, Fig. S4 *B*–*D*). MB11 outperformed the previously described PD33 antibody Fab fragment (39) both in terms of neutralization potency and breadth when compared side-by-side (Fig. 2 *D* and *E* and *SI Appendix*, Fig. S4 *A–D*), with PD33 Fab solely inhibiting PDCoV_IL121_2014_ S VSV with an IC_50_ of 611 pM (Fig. 2*E*). Collectively, these results show that MB11 is a best-in-class inhibitor which potently and broadly neutralizes a broad spectrum of DCoVs.

To prospectively evaluate the impact of possible future viral evolution on binding, we performed deep mutational scanning (DMS) of the PDCoV_IL121_2014_ RBD and assessed how all single amino acid mutations modulated recognition by five of the MBs (Fig. 2*F* and *SI Appendix,* Fig. S5 *B* and *C*). We identified a few mutations mapping to RBD loops 1 and 3 that dampen MB11 binding. However, most of these amino acid residue substitutions are associated with marked reduction of gAPN binding avidity, which would be expected to severely limit viral fitness, and/or require several nucleotide changes. Accordingly, none of these mutations are found in known PDCoV isolates with the exception of N397D which is found in PDCoV_GXCZ04-2021_ (GenBank ID: OR659139.1). The V395N substitution is also found in AvianCoV_rub035cor1_, explaining the lack of MB11 binding. MB10 showed a nearly identical escape profile to MB11 which was expected as the interface residues are the same between the two MBs. Most of the identified MB5 and MB7 escape mutations would also decrease gAPN receptor binding by two-to-three orders of magnitude and in turn viral fitness (Fig. 2*F*). Collectively, these data underscore that MB11 is a potent and broadly neutralizing DCoV inhibitor targeting the RBD and a promising countermeasure.

### MB11 Is Resilient to Various Biochemical Stresses.

**Fig. 3.**
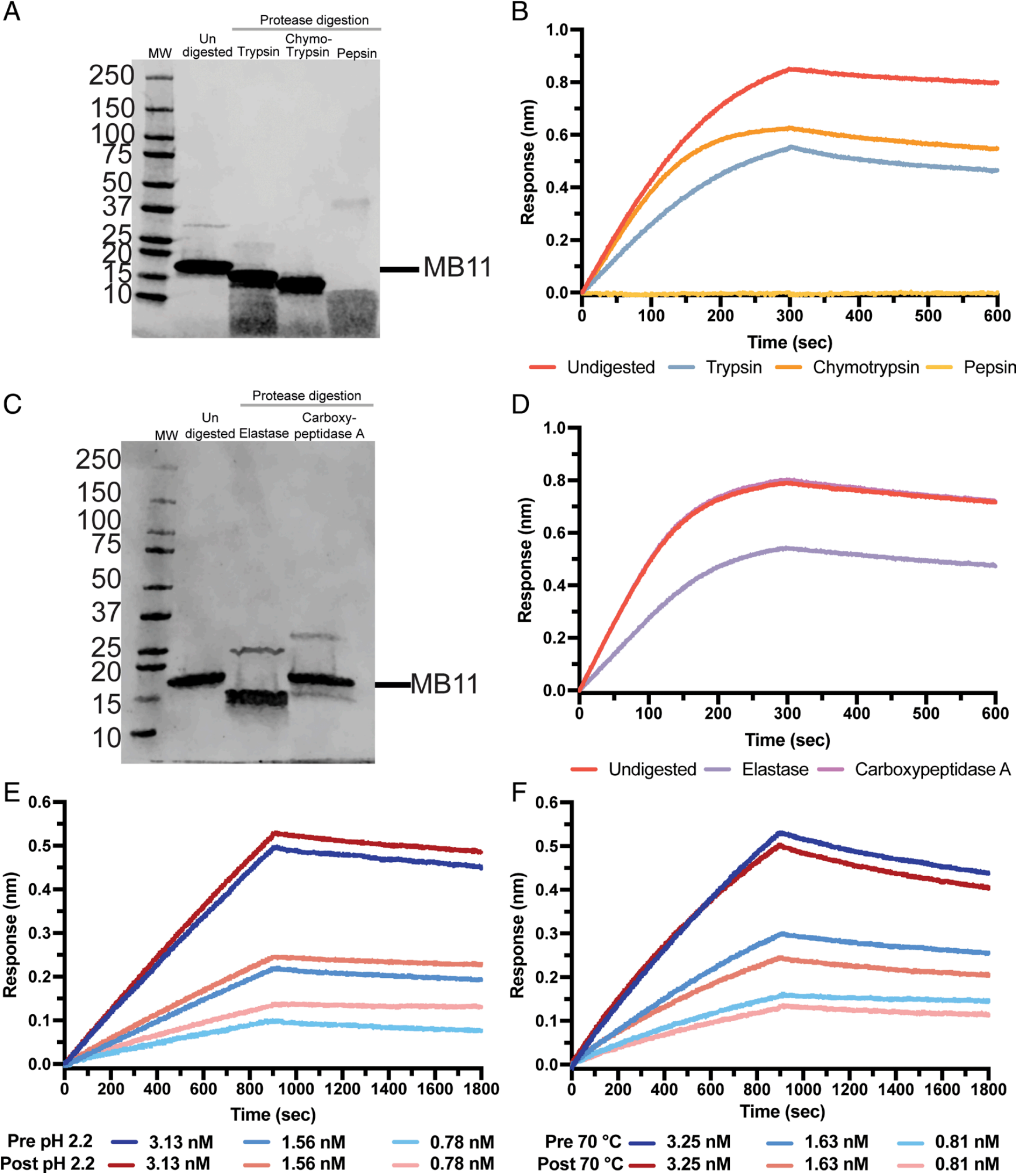
MB11 remains active after various biochemical stress. (*A*) SDS-PAGE of MB11 after 2 h incubation with trypsin, chymotrypsin, or pepsin at 37 °C at a MB11:protease ratio of 5:1 (w/w). (*B*) BLI analysis of binding of MB11 at a concentration of 25 nM, before and after protease treatment as in (*A*), to the PDCoV_IL121_2014_ RBD immobilized to streptavidin biosensors. (*C*) SDS-PAGE of MB11 after 2 h incubation with elastase or carboxypeptidase A at 37 °C at a MB11:protease ratio of 5:1 (w/w). (*D*) BLI analysis of binding of MB11 at a concentration of 25 nM, before and after protease treatment as in (*C*). (*E*) BLI analysis of binding of the PDCoV_IL121_2014_ RBD to MB11 (before and after incubation at pH 2.2 and 37 °C for 2 h) immobilized to streptavidin biosensors. (*F*) BLI analysis of binding of the PDCoV_IL121_2014_ RBD to MB11 (before and after incubation at 70 °C for 1 h) immobilized to streptavidin biosensors.

Since PDCoV is an enteropathogenic virus (35, 36), countermeasures would likely need to be delivered to the intestinal mucosa to prevent viral entry at the initial site of infection. Major proteases of the gastrointestinal tract include trypsin, chymotrypsin, pepsin, elastase, and carboxypeptidase A and B (50). We therefore set out to characterize the resistance of MB11 to various biochemical stresses in vitro to assess its biophysical stability and retention of function in various conditions. MB11 experienced minor-to-no shifts in electrophoretic mobility upon treatment with trypsin, chymotrypsin (Fig. 3*A* and *SI Appendix*, Fig. S8*A*), elastase and carboxypeptidase A (Fig. 3*C* and *SI Appendix*, Fig. S8*C*) when incubated at a 5:1 (w/w) ratio for 2 h at 37 °C and pH 7.4 (trypsin, chymotrypsin, and carboxypeptidase A) or pH 8.5 (elastase). Consequently, we observed retention of MB11 binding to the PDCoV_IL121_2014_ RBD after trypsin, chymotrypsin (Fig. 3*B* and *SI Appendix*, Fig. S8*B*), elastase, and carboxypeptidase A (Fig. 3*D* and *SI Appendix*, Fig. S8*D*) treatment, suggesting that the small shift in apparent molecular weight might have resulted from cleavage of the flexible tag (*SI Appendix*, Fig. S8*G*). However, MB11 was extensively proteolyzed by pepsin at pH 2.2 and lost binding to the PDCoV_IL121_2014_ RBD (Fig. 3 *A* and *B* and *SI Appendix*, Fig. S8 *A* and *B*). Strikingly, no appreciable reduction of MB11 binding to the PDCoV_IL121_2014_ RBD was detected after 2 h incubation at pH 2.2 before dilution into a neutral pH buffer (Fig. 3*E* and *SI Appendix*, Fig. S8*E*), showing that low pH does not irreversibly affect the integrity of MB11. Furthermore, MB11 binding to the PDCoV_IL121_2014_ RBD was retained after a 1 h incubation at 70 °C in a buffer at pH 7.4 (Fig. 3*F* and *SI Appendix*, Fig. S8*F*). Analytical size-exclusion chromatography suggests that MB11 is not aggregated post incubation at pH 2.2 or 70 °C (pH 7.4) (*SI Appendix*, Fig. S9 *A* and *B*). Collectively, these results show that MB11 is endowed with favorable biophysical properties which are ideally suited for cost-effective manufacturing and distribution and possible future use as a countermeasure.

### MB11 Inhibits Viral Entry Via Interference With Binding to the Host Receptor.

**Fig. 4.**
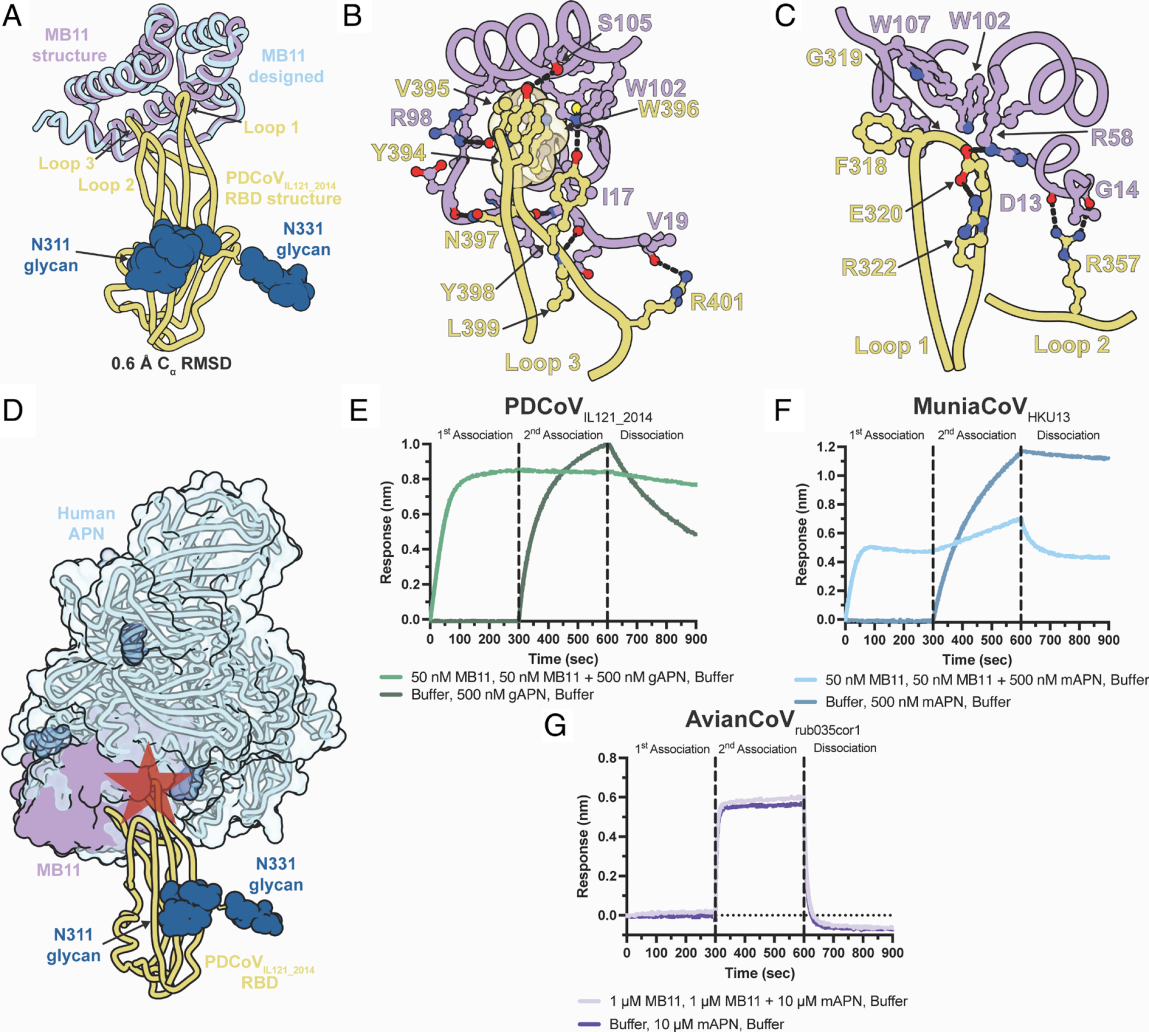
Molecular basis of MB11-mediated neutralization of divergent DCoVs. (*A*) Superimposition of the MB11-bound PDCoV_IL121_2014_ RBD (gold) from the computationally designed model (blue, predicted with AlphaFold3) and the experimental cryoEM structure (purple, PDB: 11ZV). Only one RBD is shown for clarity and the three receptor-binding loops are labeled. (*B* and *C*) Zoomed-in views of the interface between MB11 (purple) and the PDCoV_IL121_2014_ RBD (gold). Select side chains are shown as surfaces with a subset of them surrounded by semitransparent surfaces. (*D*) Structural overlay of MB11-bound (purple) and hAPN-bound (light blue, PDB: 7VPQ) PDCoV_IL121_2014_ RBD (yellow). The star indicates steric clashes between MB11 and hAPN. BLI analysis of APN-Fc binding to the PDCoV_IL121_2014_ (*E*), MuniaCoV_HKU13_ (*F*), and AvianCoV_rub035cor1_ (*G*). RBDs immobilized to the surface of streptavidin biosensors with and without prior MB11 association.

To understand the molecular basis of MB11-mediated PDCoV neutralization, we determined a cryo-EM structure of MB11 bound to the PDCoV_IL121_2014_ RBD. To aid structure determination, we used the RBD-directed Fab fragment of the noninhibitory antibody PD3 (39) and an anti-kappa light-chain nanobody (51) binding to the Fab hinge region to rigidify it, increasing the molecular weight of the complex to ~100 kDa. We obtained an asymmetric reconstruction at 2.8 Å overall resolution which we used to carry out local refinement of the MB11 and RBD region leading to a 2.8Å resolution map with improved resolvability of this region (*SI Appendix*, Fig. S10) which we used to build an atomic model of the MB11-bound PDCoV_IL121_2014_ RBD (Fig. 4*A* and *SI Appendix*, Table S3).

The MB11-bound PDCoV_IL121_2014_ RBD cryo-EM structure closely resembles the AF3 predicted structure with C_α_ RMSD of 0.6Å (PDCoV residues 307 to 417 and MB11 residues 3 to 129), underscoring the accuracy of the design (Fig. 4 *A*–*C*). MB11 is mainly alpha helical and forms a concave surface accommodating PDCoV_IL121_2014_ RBD loops 1 and 3 with more tenuous contacts with loop 2. The binding interface buries an average surface area of 800Å^2^ involving a constellation of polar interactions and shape complementarity (*SI Appendix*, Table S4). The structural data provide a molecular blueprint explaining the DMS results with G319, Y394, V395, W396, Y398, and L399 as residues with the greatest escape. Analysis of the contacts between MB11 and PDCoV_IL121_2014_ RBD shows that PDCoV G319 residue substitutions would cause steric clash and prevent MB11 association (Fig. 4*C*). PD33 and PD41 IgGs were similarly sensitive to mutations at this position which are expected to be disfavored as they are universally deleterious for receptor-binding affinity (39). Other key interactions between MB11 and PDCoV _IL121_2014_ RBD include van der Waals and pi-stacking interactions (residues Y394, V395, W396, Y398, and L399), hydrogen bonding (residues Y394, Y398, and L399), and a beta sheet augmentation of PDCoV_IL121_2014_ RBD loop 3 (Fig. 4 *B* and *C*). This extensive network of interactions likely explains the tight binding affinity of MB11 to the PDCoV_IL121_2014_ RBD. Given that MB11 binds to the receptor-binding loops which are obscured when the RBD is closed, it is expected that RBD opening is a prerequisite for MB11 engagement, as is the case for the host receptor.

To further our understanding of the broad MB11 cross-reactivity with DCoVs, we used AF3 to predict the structure of MB11 in complex with the SparrowCoV_ISU42824_ and MuniaCoV_HKU13_ RBDs (*SI Appendix*, Fig. S11). The predicted binding interfaces bury comparable surface area with the SparrowCoV_ISU42824_ RBD (866 Å^2^) and the MuniaCoV_HKU13_ RBD (815 Å^2^) to that observed in our cryoEM structure of the MB11-bound PDCoV_IL121_2014_ RBD. One notable difference is PDCoV_IL121_2014_ Y398 which is substituted to H396 for SparrowCoV_ISU42824_. The AF3 prediction of the MB11 and AvianCoV_rub035cor1_ indicated low confidence of complex formation (ipTM <0.4), consistent with our BLI results showing no detectable binding to the AvianCoV_rub035cor1_ RBD at a concentration of 50 nM (Fig. 2*C* and *SI Appendix*, Fig. S6). The AF3-predicted AvianCoV_rub035cor1_ RBD structure is quite distinct from that of the PDCoV_IL121_2014_, SparrowCoV_ISU42824_, and MuniaCoV_HKU13_ RBDs with an insertion of three residues and a predicted disulfide bond in loop 1 and overall low sequence conservation in all three loops (*SI Appendix*, Fig. S12). These features along with the aforementioned DMS data likely explain the lack of detectable MB11 binding to the AvianCoV_rub035cor1_ RBD and of neutralization of AvianCoV_rub035cor1_ S VSV.

Superimposition of the hAPN-bound and MB11-bound PDCoV_IL121_2014_ RBD structures suggests that MB11 would interfere with receptor binding competitively, concurring with the DMS data (Fig. 4*D*). To test this hypothesis experimentally, we used competition BLI which showed that MB11 interferes with APN binding to PDCoV_IL121_2014_ and MuniaCoV_HKU13_ RBDs (Fig. 4 *E* and *F* and *SI Appendix*, Fig. S13 *A* and *B*), which we cross-validated using competition ELISAs (*SI Appendix*, Fig. S14 *A* and *B*). Given that we did not detect binding of MB11 to the AvianCoV_rub035cor1_ RBD, MB11 did not block AvianCoV_rub035cor1_ RBD binding to Munia APN (Fig. 4*G* and *SI Appendix*, Fig. S13*C*). These results show that MB11 inhibits viral entry by preventing engagement of the host receptor through steric hindrance, explaining its potent neutralizing activity.

## Discussion

CoVs recurrently spillover from natural host reservoirs into humans with some most recent examples including PDCoV in 2014–2015, CCoV-HuPn-2018 in 2017–2018 (52, 53), SARS-CoV-2 in 2019 (10, 11), and MERS-CoV with cases reported in 2025 (54). These zoonotic spillovers underscore the need for vaccines and inhibitors to prevent and treat CoV infections. However, development of countermeasures broadly neutralizing SARS-CoV-2 variants and other sarbecoviruses has proven challenging (55, 56), highlighting the difficulty of designing broad spectrum coronavirus countermeasures.

We describe here five MBs recognizing the PDCoV_IL121_2014_ RBD and inhibiting PDCoV_IL121_2014_ S pseudotyped VSV entry into cells. MB11 has ultrapotent neutralizing activity, and cross-reacts with and neutralizes a panel of DCoVs sharing as little as 59% RBD amino acid sequence identity. Therefore, MB11 markedly outperforms the potency of antibodies and small molecules described against PDCoV (39–44) and is endowed with more potent and broadly neutralizing activity than sarbecovirus antibodies (57, 58). MB11 thus stands apart from most viral glycoprotein-directed antibodies, as it is not limited by the usual tradeoff between potency and breadth but instead achieves both simultaneously (57, 59).

Deep mutational scanning analysis revealed that MB11 shows a narrow escape profile with only two main positions (Y394 and V395) at which substitutions could promote evasion without a major decrease in receptor binding affinity. Only one of these mutations is present in a single PDCoV isolate explaining the observed broad PDCoV neutralizing activity. We previously showed that multimerization of SARS-CoV-2 and MERS-CoV minibinders reduced dissociation kinetics and enhanced neutralization breadth (45–47, 60). We note that MB11 exhibits remarkably potent and broad inhibitory activity as a monomer and did not require multimerization. Future evaluation of homo-oligomeric MB11 constructs bears the promise of increasing its activity even further, we propose that cocktail formulations with MB5, MB7, PD33, or PD41 would raise even higher the barrier to emergence of escape mutants given that they have distinct escape profiles (61–63).

Although the target tissue specificity for inhibiting PDCoV in humans is unclear, countermeasures would likely need to be delivered to the mucosa to protect against infection since PDCoV is enteropathogenic in pigs (34). We show that MB11 tolerates a wide range of biochemical stresses which is ideal for administration to the enteric track and withstanding its harsh environment. For mucosal delivery, MB11 could be delivered orally either using lipid nanoparticle-formulated mRNA (64) or in lyophilized form (47, 65). Alternatively, genetic fusion of MB11 to a human Fc fragment could increase half-life if systemic delivery is required. Although miniprotein inhibitors have been previously shown to have low immunogenicity and to retain efficacy after repeat dosing in mouse models (66, 67), future studies are needed to assess the immune response to monovalent and multivalent miniproteins in humans. De *novo* design of miniprotein inhibitors has yielded cost-effective and potent viral neutralizers enabling to combat emerging CoVs (45–47) and to prepare for possible future pandemics.

## Materials and Methods

### Cell Lines.

HEK293T cells (ATCC, Cat# CRL-11268) were cultured in Dulbecco’s Modified Eagle Medium (DMEM) supplemented with 10% fetal bovine serum (FBS) (Cytiva) and 1% penicillin-streptomycin (Thermo Fisher Scientific) at 37 °C and 5% CO_2_. Expi293F cells (Thermo Fisher Scientific, Cat# A14527) were cultured in Expi293F expression medium (Thermo Fisher Scientific), shaking at 130 rpm, 37 °C, and 8% CO_2_.

### PDCoV_IL121_2014_ RBD MB Design.

PDCoV_IL121_2014_ RBD MBs were designed using BindCraft (48). The following target residues from PDCoV_IL121_2014_ RBD were chosen: 316 to 323, 357, 389 to 403 based on the APN receptor binding motif (37). MBs were filtered based on default BindCraft metrics. Filtering resulted in 101 MB designs. From the 101 MBs, 17 MBs were selected for expression and purification based on AlphaFold 3 metrics ipTM (>0.7) and pTM (>0.8) values and visual assessment of the MB, PDCoV_IL121_2014_ RBD binding interface from the AF3 predicted structure. BindCraft ranked MBs 1 to 11, 20, 21, 29, 50, 52, and 95 were chosen for experimental validation. An 8× His followed by an AVI tag (N term constructs) or AVI followed by an 8× His tag (C term constructs) with a thrombin cleavage site (LVPRGS) in between were added to each MB construct. Purification and biotinylation tags were added to the N or C termini of the MBs based on the predicted MB, PDCoV_IL121_2014_ RBD AF3 structure.

### Production and Purification of PDCoV_IL121_2014_ RBD MBs.

Seventeen PDCoV_IL121_2014_ RBD MB genes were cloned by Genscript into a pET29b(+) vector using NdeI and XhoI restriction sites. MBs were transformed into BL21(DE3)pLysS cells (Promega, Cat# L1195) using Kanamycin (50 µg/mL) as the selection antibiotic. Single colonies were selected and grown overnight. Initial screening of MB was performed in 100 mL LB media. MBs that showed binding to PDCoV_IL121_2014_ S1-Fc were then expanded to 700 mL LB media and grown at 37 °C until OD_600_ reached 0.6 to 0.8. Expression of MBs was induced by addition of IPTG to a final concentration of 1 mM and cultures were grown overnight at 15 °C. Cultures were harvested at 7,000×*g* for 10 min at 4 °C by centrifugation. The pellets were resuspended in 8 mL of lysis buffer (50 mM HEPES pH 8 and 300 mM NaCl) with the addition of 1 mg/mL lysozyme, 10 µg/mL DNAse, and 1 mM PMSF per gram of bacterial cell pellet. Resuspended pellets were incubated at room temperature rotating at 15 rpm for 30 min. Cells were lysed by passing the lysate three times at a pressure of 15,000 psi through an emulsiflex C3 homogenizer (Avisten). The lysates were then centrifuged at 30,000×*g* at 4 °C for 30 min and the supernatant was incubated with 1 mL of pre-equilibrated Talon resin (Takara) for 30 min rotating at 15 rpm. The resin was subsequently poured into a gravity column (Cytiva) and washed with wash buffer (50 mM HEPES pH 8, 300 mM NaCl, and 5 mM Imidazole pH 8) until the A_280_, measured by a Nanodrop 2000 (Thermo Fisher Scientific) was below 0.01. MBs were eluted from the resin with 20 to 30 column volumes of elution buffer (50 mM HEPES pH 8, 300 mM NaCl, and 300 mM Imidazole pH 8). MBs were concentrated using an Amicon 10 kDa MWCO ultracentrifugal filter (Millipore) and run on an Superdex 75 increase 10/300 GL (Cytiva). The resulting A_280_ peaks were collected in 0.5 mL fractions and analyzed with SDS-PAGE. MBs were concentrated, aliquoted, frozen using liquid nitrogen, and stored at −80 °C until use.

### Transformation of DH10B cells and Purification of DNA for Mammalian Cell Transfection.

Plasmids (*SI Appendix*, Table S5) were transformed into DH10B cells (Thermo Fisher Scientific) following the manufacturer instructions and using 100 µg/mL of carbomycin as the selection antibiotic. Single colonies were picked and grown overnight in 500 mL LB supplemented with 100 µg/mL ampicillin. The plasmids were purified using a Mega Kit (QIAGEN) following the manufacturer instructions.

### Transfection of Expi293F Cells for Recombinant Protein Expression.

Expi293F cells were grown at 37 °C with 8% CO_2_ as described above to a density of 3.0 × 10^6^ cells/mL and were transfected using the Expifectamine 293 transfection kit (Thermo Fisher Scientific, Cat# A14525) following the manufacturer instructions. Cell supernatant was clarified by centrifugation at 3,500 rpm for 20 min four d post transfection and filtered with a 0.22 µm filter.

### Expression and Purification of PDCoV_IL121_2014_ S1-Fc.

PDCoV_IL121_2014_ S_1_-Fc (*SI Appendix*, Table S5) was expressed in Expi293F cells as described above. The supernatant was incubated with 2 mL of Mab select resin (Cytiva) pre-equilibrated in wash buffer (50 mM Tris pH 7.4 and 150 mM NaCl) for 1 h shaking at 160 rpm. The resin was washed with wash buffer until the A_280_ from the eluate was less than 0.01. PDCoV S_1_-Fc was eluted from the resin with 10 CV of elution buffer (0.1 M citric acid pH 3 and 150 mM NaCl) into 10 mL of neutralization buffer (1 M Tris pH 9 and 150 mM NaCl). The eluates were concentrated using a 50 kDa MWCO ultracentrifugal filter (Amicon) and injected onto a Superose 6 Increase 10/300 GL (Cytiva). The resulting peaks were analyzed via SDS-PAGE for purity and fractions were pooled and concentrated using a 50 kDa MWCO ultracentrifugal filter (Amicon). Protein was aliquot and snap frozen in liquid nitrogen and stored at −80 °C until use.

### Expression and Purification of DCoV RBDs.

PDCoV_IL121_2014_, SparrowCoV_ISU42824_, MuniaCoV_HKU13_, and AvianCoV_rub035cor1_ RBDs (*SI Appendix*, Table S5) were expressed in Expi293F cells and harvested as described above. HEPES pH 8 was added to the supernatant to a final concentration of 50 mM and incubated with 1 mL of Ni Sepharose Excel resin (Cytiva) pre-equilibrated in wash buffer (50 mM HEPES pH 8, 300 mM NaCl, 10 mM imidazole) for 1 h shaking at 160 rpm. The resin was washed with wash buffer until the A_280_ from the eluate was less than 0.01. The RBDs were eluted from the resin with 15 CV of elution buffer (50 mM HEPES pH 8, 300 mM NaCl, 300 mM imidazole). The eluate was concentrated using a 10 kDa MWCO ultracentrifugal filter (Amicon) and injected onto a Superdex 75 increase 10/300 GL (Cytiva). The resulting peak was analyzed via SDS-PAGE for purity and fractions were pooled and concentrated using a 10 kDa MWCO ultracentrifugal filter (Amicon). Protein was aliquot and snap frozen in liquid nitrogen and stored at −80 °C until use.

### Expression and Purification of APN Ectodomains.

The APN ectodomains-Fc (*SI Appendix*, Table S5) were expressed and purified as described above for PDCoV_IL121_2014_ S_1_-Fc.

### Expression and Purification of PD3 Fab.

The PD3 Fab (*SI Appendix*, Table S5) was expressed and purified as described above for DCoV RBDs except the PD3 Fab was purified by a Superdex 200 Increase 10/300 GL after Ni Sepharose Excel purification.

### Expression and Purification of Anti-Human Kappa Chain Nanobody.

The anti-human kappa light chain nanobody was expressed and purified as previously described (39).

### Production of DCoV S Pseudotyped VSV.

PDCoV_IL121_2014_ (39), SparrowCoV_ISU42824_, MuniaCoV_HKU13_, and AvianCoV_rub035cor1_ S plasmids (*SI Appendix*, Table S5) were purified as described above. 8.5 × 10^6^ HEK293T cells were seeded in 100 mm BioCoat Poly-D-Lysine dishes (Corning). Cells were incubated for 18 h, washed twice with 5 mL of growth medium, and transfected with 24 µg of the respective S plasmid and 60 µL Lipofectamine 2000 following the manufacturer protocol (Thermo Fisher Scientific, Cat# 11668019). 3 mL of transfection mixture and 7 mL of growth medium was added to the cells. Cells were incubated for 18 to 21 h, washed four times with 5 mL of DMEM, and infected with 300 µL VSV (G*ΔG-luciferase) pseudotyped with VSV G in 10 mL of DMEM for 2 h. Cells were washed four times with DMEM and 400 µL of anti-VSV G antibody (I1-mouse hybridoma supernatant, ATCC CRL-2700) was added to 10 mL of DMEM. Cells were incubated for 24 h and the supernatant was clarified by centrifugation at 4,200 rpm for 10 min and filtered using a 0.45 µm syringe filter. Pseudotyped S VSV was concentrated 25× with a 100 kDa MWCO ultracentrifugal filter (Amicon), aliquot, and stored at −80 °C until use.

### Biolayer Interferometry.

BLI assays were performed on an Octet Red (Sartorius) instrument at 30 °C and 1,000 rpm. Biosensors were hydrated in 10× kinetics buffer (Sartorius) for 10 min prior to use. Sensorgrams were adjusted by subtraction of a reference loaded biosensor dipped into 10× kinetics buffer for association and dissociation phases.

For MB binding screening to PDCoV_IL121_2014_ S_1_-Fc, MBs were immobilized to HIS1K biosensors (Sartorius, Cat# 18-5120) at 10 µg/mL in 10× kinetics buffer until a 1 nm shift was reached. The biosensors were subsequently dipped into 10× kinetics buffer for 60 s before association with 500 nM PDCoV_IL121_2014_ S_1_-Fc for 300 s, followed by dissociation into 10× kinetics buffer for 300 s.

For K_D_ determination of MB11 and PDCoV_IL121_2014_ RBD, biotinylated MB11 was loaded onto SA biosensors (Sartorius, Cat# 18-5019) at 5 µg/mL in 10× kinetics buffer until a 1 nm shift was reached and dipped into 10× kinetics buffer for 60 s. MB11 loaded biosensors were associated with 1,250 pM to 313 pM, serially diluted twofold, PDCoV_IL121_2014_ RBD in 10× kinetics buffer for 1,300 s followed by dissociation into 10× kinetics buffer for 3,400 s. The resulting association, dissociation, and K_D_ values were calculated with ForteBio data analysis software with a 1:1 binding model and global fit of curves.

For measurement of MB11 DCoV RBD binding, MB11 was immobilized to SA biosensors as described above. MB11 loaded biosensors were associated with 50 nM of PDCoV_IL121_2014_, SparrowCoV_ISU42824_, MuniaCoV_HKU13_, or AvianCoV_rub035cor1_ RBD for 300 s, followed by dissociation into 10× kinetics buffer for 300 s.

For K_D_ determination of MB11 with SparrowCoV_ISU42824_ and MuniaCoV_HKU13_ RBD, biotinylated MB11 was loaded onto SA biosensors at 2 µg/mL in 10× kinetics buffer until a 1 nm shift was reached then dipped into 10× kinetics buffer for 60 s. MB11 loaded biosensors were associated with 49.4 nM to 0.203 nM, serially diluted threefold, SparrowCoV_ISU42824_ RBD in 10× kinetics buffer for 1,200 s followed by dissociation into 10× kinetics buffer for 300 s. For MuniaCoV_HKU13_ RBD K_D_ determination, MB11 loaded biosensors were associated with 233 nM to 0.960 nM MuniaCoV_HKU13_ RBD, serially diluted threefold, for 900 s followed by dissociation into 10× kinetics buffer for 300 s. The resulting K_D_ values were calculated with ForteBio data analysis software with a steady state binding model fit of R_eq_ versus SparrowCoV_ISU42824_ or MuniaCoV_HKU13_ RBD concentration.

MB11 binding to PDCoV_IL121_2014_ RBD was assessed by BLI after digestion by trypsin, chymotrypsin, pepsin, carboxypeptidase A, or elastase. Biotinylated PDCoV_IL121_2014_ RBD was loaded onto SA biosensors at 5 µg/mL in 10× kinetics buffer until a 1 nm shift was reached. Biosensors were blocked with 50 µg/mL Biocytin (Thermo Fisher Scientific, Cat# B1592) in PBS for 300 s, followed by washing in PBS for 300 s, and reequilibrating into 10× kinetics buffer for 60 s. PDCoV_IL121_2014_ RBD loaded biosensors were associated with 25 nM of undigested or digested MB11 for 300 s, followed by dissociation into 10× kinetics buffer for 300 s.

MB11 binding was assessed after temperature incubation at 70 °C for 1 h or pH 2.2 incubation at 37 °C for 2 h. Biotinylated MB11 was loaded onto SA biosensors as described above and MB11 loaded biosensors were associated with 3.25 nM to 0.81 nM (temperature incubation) or 2.5 to 0.63 nM (pH 2.2 incubation), serially diluted twofold, PDCoV_IL121_2014_ RBD in 10× kinetics buffer for 900 s followed by dissociation in 10× kinetics buffer for 900 s.

For APN and MB11 competition assays with DCoV RBD, biotinylated DCoV RBD was loaded onto SA biosensors as described above and blocked with 50 µg/mL Biocytin (Thermo Fisher Scientific) in PBS for 300 s, followed by washing in PBS for 300 s. RBD loaded biosensors were dipped into 10× kinetics buffer for 60 s, followed by association with or without 50 nM or 1 µM MB11 in 10× kinetics buffer for a 300 s first association. Biosensors were then dipped into 500 nM or 10 µM APN for a 300-s second association, followed by a 300 s dissociation into 10× kinetics buffer.

### Neutralization Assays.

For PDCoV_IL121_2014_ and SparrowCoV_ISU42824_ S pseudotyped VSV neutralizations, 7 × 10^6^ HEK293T cells were seeded into 100 mm plates (Falcon) in DMEM supplemented with 10% FBS and 1% penicillin-streptomycin and incubated for 24 h. HEK293T cells were washed twice with Opti-MEM (Thermo Fisher Scientific) and transfected with 8 µg of full-length gAPN or sparrow APN (*SI Appendix*, Table S5) and 30 µL of Lipofectamine 2000 following the manufacturer protocol (Thermo Fisher Scientific). Cells were washed with PBS (Cytiva) 5 h post transfection, trypsinized with 400 µL 0.25% trypsin (Thermo Fisher Scientific), and resuspended in growth medium. 40,000 cells/well were seeded into 96-well plates (MB5, 7, and 8) and 500,000 cells/well into 12-well plates (MB10 and 11, PD3 Fab, and PD33 Fab) coated with poly-L-lysine (Thermo Fisher Scientific) and incubated overnight. 80 µM MB5, 7, and 8 were serial diluted 1:3 in half-area 96-well plates followed by a 1:2 dilution with pseudotyped S VSV. 2 µM MB10 and MB11 were serial diluted 1:12 (PDCoV_IL121_2014_ S pseudotyped VSV neutralizations) or 5 µM MB10 and MB11 1:12 or 1:18 (SparrowCoV S pseudotyped VSV neutralizations) in 12-well plates, followed by 1:2 dilution with pseudotyped S VSV. 1.5 µM PD33 Fab was serial diluted 1:24 (PDCoV_IL121_2014_ S pseudotyped VSV neutralizations) or 20 µM (SparrowCoV S pseudotyped VSV neutralizations), followed by 1:2 dilution with pseudotyped S VSV. MB and pseudotyped S VSV were incubated for 30 min at room temperature and 40 µL (MB5, 7, and 8) or 500 µL (MB10 and 11) were transferred to cells, and incubated for 2 h. After incubation, cells were supplemented with 40 µL (MB5, 7, and 8) or 500 µL (MB10 and 11) of DMEM supplemented with 20% FBS and 2% penicillin-streptomycin and incubated for 22 h. After incubation, cells were treated with 40 µL (MB5, 7, and 8) or 500 µL (MB10 and 11) of One-Glo-EX substrate (Promega) and incubated at 130 rpm and 37 °C in the dark for 5 min.

For MuniaCoV_HKU13_ S pseudotyped VSV neutralizations, HEK293T cells were transiently transfected with Munia APN (mAPN) (*SI Appendix*, Table S5) as described above. 150,000 cells/well were seeded into 48-well plates coated with poly-L-lysine (Sigma Aldrich) and incubated overnight at. 80 µM MB11 was serial diluted 1:3 in 48-well plates, followed by 1:2 dilution with pseudotyped S VSV. MB and pseudotyped S VSV were incubated as described above, 150 µL was transferred to cells, and incubated for 2 h. After incubation, cells were supplemented with 150 µL of DMEM supplemented with 20% FBS and 2% penicillin-streptomycin and incubated for 22 h. After incubation, cells were treated with 150 µL of One-Glo-EX substrate (Promega) and incubated at 130 rpm and 37 °C in the dark for 5 min.

For AvianCoV_rub035cor1_ S pseudotyped VSV neutralization assays, HEK293T cells were transfected with mAPN as described above. Neutralization of pseudotyped S VSV was performed as described for the (MB5, 7, and 8) neutralizations except 80 µM MB11 was serial diluted 1:3.

Relative luciferase units (RLU) were read with a BioTek Neo2 plate reader. RLU were normalized, to determine % neutralization, by RLU from cells treated (0% neutralization) and untreated (100% neutralization) with pseudotyped S VSV in Prism (GraphPad, v10, https://www.graphpad.com). A nonlinear regression curve fit and log inhibitor vs. normalized response, variable slope equation was used to determine IC_50_ values for two technical replicates and four biological replicates.

### Quantification of MB10 and 11 Binding to DCoV RBDs Using Yeast Display.

We constructed a yeast-display library containing RBDs from all known DCoVs reported in our previous phylogenetic analysis (39). Each RBD was represented by multiple barcodes in the library reflecting internal pseudoreplication. Minibinders were incubated with the pan-DCoV library at five concentration points from 10,000 to 0.0256 mg/mL via fivefold dilutions, plus a zero minibinder concentration. Approximately 1 OD*mL of yeast library was incubated and labeled at each serial dilution. For each concentration, approximately 1 million RBD+ cells were sorted and binned from low to high binding (*SI Appendix*, Fig. S5*A*). Barcodes were counted within each FACS bin via Illumina sequencing, and EC50s were calculated based on the distribution of sequencing counts across sort bins and minibinder concentrations. The full computational pipeline is available from GitHub: https://github.com/tstarrlab/PD-CoV_breadth_minibinder

### Deep Mutational Scanning of PDCoV_IL121_2014_ RBD MB Escape.

Deep mutational scanning using a PDCoV RBD mutant library was performed as previously described (39). Briefly, a yeast-displayed PDCoV RBD site-saturation mutagenesis library was grown and induced for expression, and 1 OD*mL of cells were labeled for one h at 19 °C in 1 mL with a concentration of minibinder equal to the EC90 determined from pilot binding assays of the minibinder to yeast-displayed wildtype PDCoV RBD. In parallel, 0.5 OD*mLof the wildtype parental PDCoV RBD yeast-display strain were incubated in 100 μL minibinder at the matched EC90 concentration or 1/10th the concentration for FACS gate setting. Cells were washed, incubated with 1:100 FITC-conjugated chicken anti-Myc (Immunology Consultants CMYC-45F) to label for RBD surface expression and 1:200 PE-conjugated streptavidin (Invitrogen S866) to label for bound minibinder.

Minibinder-escape cells in each library were selected via FACS on a BD FACSAria II. FACS selection gates were drawn to capture approximately 50% of yeast expressing the wildtype PDCoV RBD labeled at the 1/10th EC90 minibinder concentration (*SI Appendix*, Fig. S5*B*). For each sample, 4 million RBD+ cells were processed on the sorter, collecting all cells in the minibinder-escape cells. Sorted cells were grown overnight in SD-CAA media, plasmid purified, and barcodes PCR amplified and Illumina sequenced. In parallel plasmid samples were purified and sequenced from 30 OD*mL of unsorted library cultures to establish presort barcode frequencies. Demultiplexed Illumina barcode reads were matched to library barcodes using dms_variants (version 0.8.9), and escape fraction computed as previously described. Final per-mutant escape fractions were calculated independently for separate DMS libraries (*SI Appendix*, Fig. S5*C*). The full computational pipeline is available from GitHub: https://github.com/tstarrlab/PD-CoV_MAP_minibinder.

### Protease Digestion of MB11 and Analysis of Digestion Sites.

25 µg of MB11 at 0.25 mg/mL was incubated with trypsin (Thermo Fisher Scientific, Cat# 90057), chymotrypsin (Thermo Fisher Scientific, Cat# 90056), pepsin (Fisher Scientific, Cat# AAJ6167903), elastase (Promega, Cat# V1891), or carboxypeptidase A (Sigma-Aldrich, Cat# SAE0046) at a 5:1 (w/w) ratio at 37 °C for 2 h. Trypsin, chymotrypsin, and carboxypeptidase A digests were performed in 50 mM Tris pH 7.4 and 150 mM NaCl while pepsin digests were performed in 50 mM glycine pH 2.2 and 150 mM NaCl and elastase digests in 50 mM Tris pH 8.5 and 150 mM NaCl. Binding to PDCoV_IL121_2014_ RBD was measured by BLI as described above. Digestion was measured by SDS-PAGE. PeptideCutter (68) was used with default settings to predict trypsin, chymotrypsin, and pepsin cleavage sites in MB11. ProsperousPlus (69) was used, with a prediction score threshold of 0.7, to predict elastase and carboxypeptidase A cleavage sites.

### Biochemical Stress Test of MB11.

MB11 was tested for thermal stability by 70 °C incubation of 100 µg MB11 at 0.25 mg/mL in 50 mM Tris pH 7.4 and 150 mM NaCl for 1 h. 25 µg MB11 at 0.25 mg/mL was incubated in 50 mM glycine pH 2.2 and 150 mM NaCl at 37 °C for 2 h. Binding to PDCoV_IL121_2014_ RBD after temperature or pH incubation was tested by BLI as described above. Identical conditions were used to test for aggregation by analytical size exclusion chromatography. MB11 was injected onto a S75 increase 10/300 GL (Cytiva) in 50 mM Tris pH 7.4 and 150 mM NaCl.

### Cryo-EM Sample Preparation and Data Collection.

PDCoV_IL121_2014_ RBD was complexed with PD3 Fab at a 1.2× molar excess of RBD for 1 h at room temperature. Unbound RBD was removed by centrifugation with a 30 kDa ultracentrifugal filter (Amicon). PDCoV_IL121_2014_ RBD, PD3 Fab complex was mixed with a 4× molar excess of MB11 and 1.2× molar excess of anti-human kappa light chain nanobody and incubated for 1 h at room temperature. Unbound anti-human kappa light chain nanobody and MB11 were removed by centrifugation with a 50 kDa ultracentrifugal filter (Amicon). 3 µL of 3 mg/mL PDCoV_IL121_2014_ RBD/MB11/PD3 Fab/nanobody complex was mixed with 0.3 µL 30 mM CHAPSO before 3 µL was added to a glow discharged R2/2 UltrAuFoil grid (70). The grid was blotted with a blot force of 0 and blot time of 6 s at 100% humidity and 22 °C before the grid was plunge frozen using a Vitrobot Mark IV (Thermo Fisher Scientific). Data were collected using a FEI Titan Krios transmission electron microscope operating at 300 kV with a Gatan K3 direct detector and Gatan Quantum GIF energy filter that was operated in zero-loss energy mode and a slit width of 20 eV. Leginon (71) was used to perform automated data collection at a 105,000 nominal magnification, 0.829 Å pixel size, and total dose exposure of 53 e/Å^2^ in counting mode. 10,236 movies were collected with a defocus range of −0.5 to −1.5 µm. Each movie was divided into 100 frames at 40 ms each.

### Cryo-EM Data Processing, Refinement, and Model Building.

Motion correction, contrast transfer function parameter estimation, automatic particle picking, and extraction was performed using Warp (72). Particles were extracted with a 168 pixel box size and a 1.67 Å binned pixel size. Particles were selected and used to perform ab initio reconstruction after 2D classification in cryoSPARC (v4.7.1, https://cryosparc.com) (73). Heterogeneous refinement was performed using the ab initio reconstruction as the initial model. A nonuniform refinement (74) was performed before training Topaz particle picker with the selected set of particle images (75). The Topaz picked particles were extracted and sorted using 2D classification and heterogeneous refinement before refinement using nonuniform refinement in cryoSPARC. Particle stacks from Warp and Topaz were combined and duplicate particles were removed with a cutoff distance of 90 Å. A nonuniform refinement was performed on the combined particle stacks in cryoSPARC. Particle data were transferred from cryoSPARC to RELION (v3.0, https://www3.mrclmb.cam.ac.uk/relion) (76, 77) using the pyem program package (78). Bayesian polishing in RELION (79). The particles were transferred back to cryoSPARC and 2D classification, heterogenous refinement, nonuniform refinement with per-particle defocus refinement were performed to yield a 2.8 Å reconstruction with 512,440 particles. A PDCoV_IL121_2014_ RBD, MB11 mask was created in ChimeraX and imported into cryoSPARC with a 5 pixel soft padding width to carry out local refinement which yielded a 2.8Å reconstruction with improved MB11 resolvability.

Local resolution estimation, sharpening, and filtering were performed using cryoSPARC. ChimeraX (https://www.cgl.ucsf.edu/chimerax/) (80), Coot (https://www2.mrc-lmb.cam.ac.uk/personal/pemsley/coot/) (81), and ISOLDE (https://tristanic.github.io/isolde/) (82) were used to fit the atomic models into the cryo-EM maps. The models were refined and validated using Phenix (https://www.phenix-online.org/) (83), Molprobity (http://molprobity.biochem.duke.edu/) (84), and Privateer (https://github.com/glycojones/privateer) (85). Interface residues were analyzed with PISA (86).

### Competition ELISAs.

MB11, gAPN-Fc competition was measured by ELISA. 50 µL of biotinylated PDCoV_IL121_2014_ RBD at 30 µg/mL was immobilized to 384-well neutravidin plates (Thermo Fisher Scientific, Cat# 15400) overnight at 4 °C. Plates were washed 4× with tris buffered saline and tween-20 (TBST-50 mM Tris pH 7.5, 150 mM NaCl, and 0.1% tween-20) with a plate washer (BioTek). Plates were blocked with 30 µL of casein blocking buffer (Thermo Fisher Scientific) for 1 h at 37 °C. Plates were washed 4× with TBST. For gAPN-Fc EC_50_ determination, gAPN-Fc was serial diluted 1:3 in TBST from a starting concentration of 2.5 µM or 1.25 µM and 30 µL was transferred to plates with immobilized PDCoV_IL121_2014_ RBD. The plates were incubated for 1 h at 37 °C before washing 4× with TBST. After washing, goat anti-human IgG conjugated to horseradish peroxidase (Thermo Fisher Scientific, Cat# A18823) was diluted 1:5000 in TBST and 30 µL was transferred to plates with immobilized PDCoV_IL121_2014_ RBD. Plates were incubated with IgG for 1 h at 37 °C and washed 4× with TBST. 30 µL of TMB (SeraCare) was added to ELISA plates and incubated at room temperature for 2 min before addition of 30 µL 1 N HCl. Plates were read at 405 nm on a BioTek Neo2 plate reader. The resulting curves were graphed in Prism (GraphPad) and fit with a nonlinear regression, sigmoidal 4PL x is concentration equation. The resulting EC_50_ value was calculated from the fitted curve. Competition assays were performed at 15 nM gAPN-Fc (9× the calculated EC_50_). MB, gAPN-Fc competition was performed at a starting concentration of 40 µM (MB5), 40 or 80 µM (MB7), 60 or 80 µM (MB8), and 5 or 2.5 µM (MB10 and 11), serial diluted 1:3 and diluted 1:2 with 30 nM gAPN-Fc. 30 µL of MB, gAPN-Fc mixture was transferred to biotinylated PDCoV_IL121_2014_ RBD coated neutravidin plates and incubated at 37 °C for 1 h. Plates were washed, incubated with goat anti-human IgG conjugated to horseradish peroxidase, and read as described above. The resulting curves were graphed in Prism (GraphPad) and fit with a nonlinear regression, sigmoidal 4PL× is concentration equation. Constraints were applied where the bottom value constrained to the A_405_ value with PDCoV_IL121_2014_ RBD coated wells incubated with TBST and top value constrained to PDCoV_IL121_2014_ RBD coated wells incubated with 15 nM gAPN-Fc. The IC_50_ values were calculated from the fitted curves.

## Supplementary Material

Appendix 01 (PDF)

## Data Availability

Cryo-EM maps and models of the PDCoV, MB11 structure have been deposited in the EMDB and PDB with the accession numbers EMD-76233 (87) and PDB: 11ZW (88) and EMD-76232 (89) and PDB: 11ZV (90) (local refinement) respectively. All other data are included in the manuscript and/or *SI Appendix*.
