# Supplementary material for: Computational design of an ultrapotent deltacoronavirus miniprotein inhibitor
Source: Proc Natl Acad Sci U S A. 2026 Apr 29;123(18):e2533456123. doi: 10.1073/pnas.2533456123 (PMC13142991; doi:10.1073/pnas.2533456123)
Supplement: Supplementary file 1 — Appendix 01 (PDF) [file pnas.2533456123.sapp.pdf]

Supporting Information for
Computational design of an ultrapotent deltacoronavirus miniprotein inhibitor

Nathan G. Avery[1], Courtney N. Yoshiyama[1], Ashley L. Taylor[2], Young-Jun Park[1,3], Daniel Asarnow[1], Lisa Perruzza[4], Jack T. Brown[1], Davide Corti[4], Fabio Benigni[4], Tyler N. Starr[2] and David Veesler[1,3,†]

[†]**Correspondance:** David Veesler
Email: dveesler@uw.edu

This PDF file includes:

Supporting text
Figures S1 to S14
Tables S1 to S5
SI References

Figures

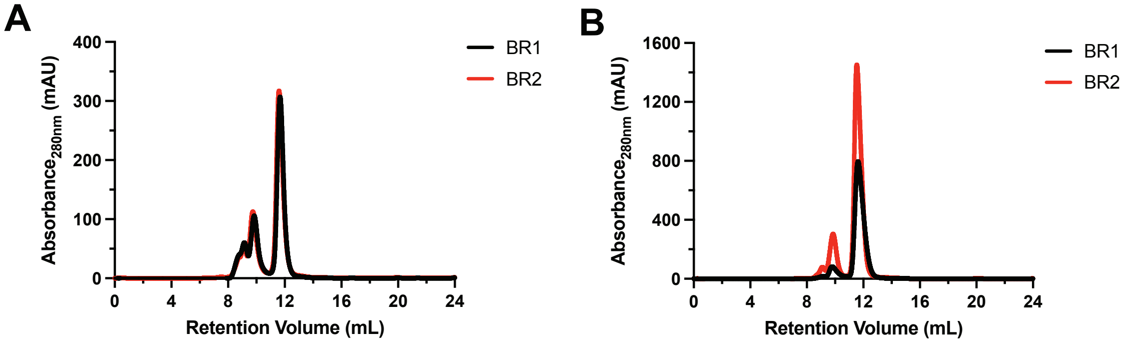

Fig. S1., related to Figure 1: Size exclusion chromatography of MB10 and MB11. A-B, MB10 (A) and MB11 (B) were purified using a Superdex 75 increase 10/300 GL (Cytiva) equilibrated in TBS after IMAC purification. Two independent batches of protein were analyzed for protein aggregation and monodispersity. BR1 and BR2: biological replicates 1 and 2, respectively.

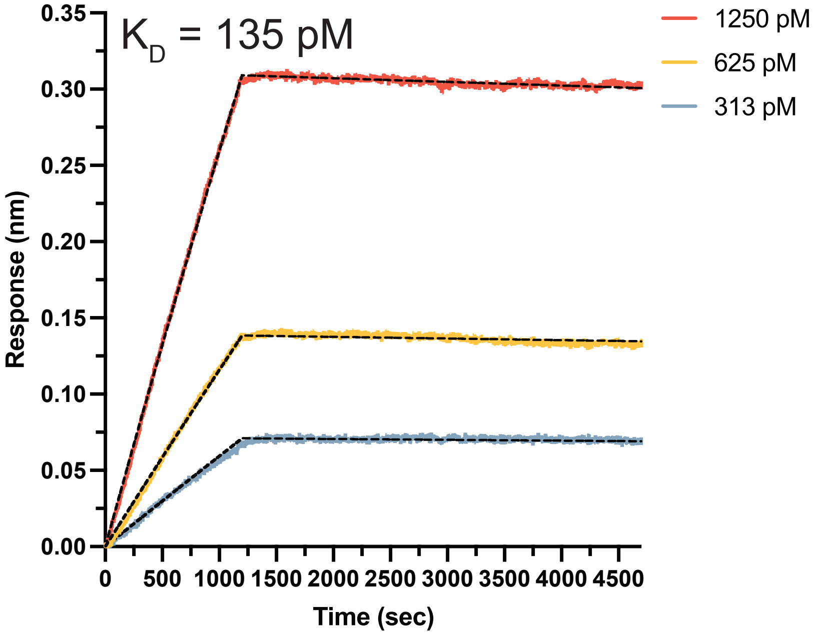

Fig. S2., related to Figure 1: Biological replicate 2 of biolayer interferometry analysis of the PDCoV$_{IL121_2014}$ RBD binding to MB11. Biotinylated MB11 at a concentration of 5 µg/mL was loaded onto streptavidin biosensors until a 1 nm shift was reached. Binding of the PDCoV$_{IL121_2014}$ RBD at concentrations of 1250, 625, and 313 pM in 10x kinetics buffer (Sartorius) was monitored for 1,300 seconds, followed by dissociation into 10x kinetics buffer for 3,400 seconds. The data was fit to a global 1:1 binding model (dashed black line) to determine the equilibrium dissociation constant (K_D). Two biological replicates with two batches of each protein were performed.

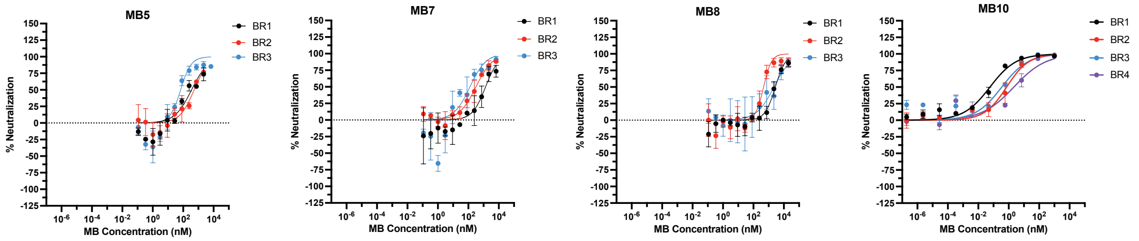

Fig. S3., related to Figure 1: Neutralization of PDCoV$_{IL121_2014}$ S pseudotyped VSV mediated by a panel of MBs. Dose-dependent MB-mediated neutralization of VSV pseudotyped with PDCoV$_{IL121_2014}$ S using HEK293T target cells transiently transfected with galline APN (gAPN). Each data point represents the average of three technical replicates. SD are shown as error bars. Three to four different batches of protein and pseudoviruses were used (BR1-4).

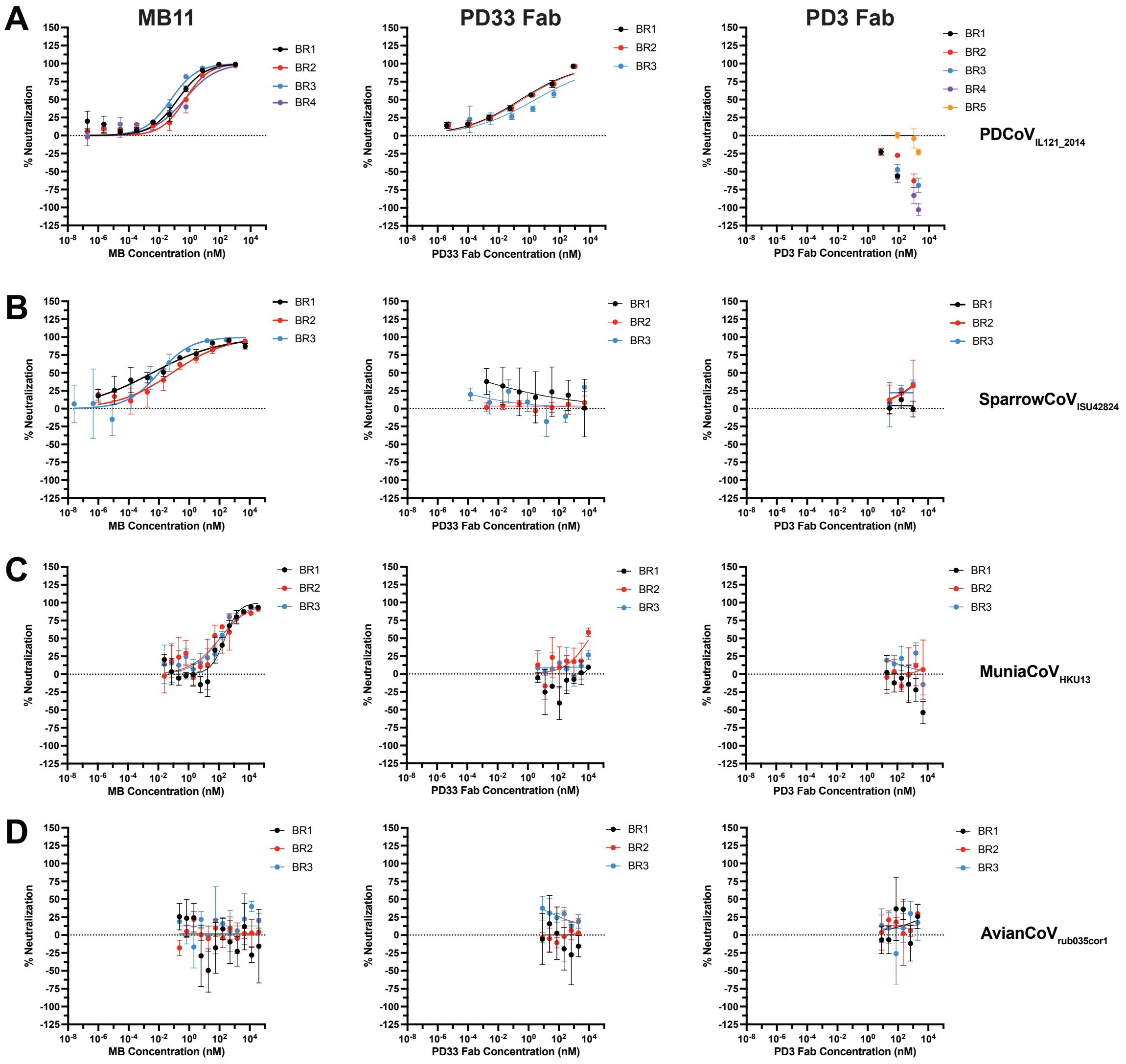

Fig. S4., related to Figures 1 and 2: Neutralization of DCoV S pseudotyped VSV mediated by MB11 and Fabs. A-D, Dose-dependent MB11-, PD33 Fab-, and PD3 Fab-mediated neutralization of VSV pseudotyped with PDCoV$_{IL121_2014}$ (A), SparrowCoV$_{ISU42824}$ (B), MuniaCoV$_{HKU13}$ (C), and AvianCoV$_{rub035cor1}$ (D) S using HEK293T target cells transiently transfected with gAPN (PDCoV$_{IL121_2014}$), sparrow APN (sAPN, SparrowCoV$_{ISU42824}$), or munia APN (mAPN, MuniaCoV$_{HKU13}$ and AvianCoV$_{rub035cor1}$). For PDCoV$_{IL121_2014}$ and SparrowCoV$_{ISU42824}$ S VSV neutralization, each point represents the average of two technical replicates. For MuniaCoV$_{HKU13}$ and AvianCoV$_{rub035cor1}$ S VSV neutralization, each point represents the average of three technical replicates. SD are shown as error bars. For MB11 neutralization, three different batches of protein and four different batches of pseudotyped S VSV were used. For PD33 and PD3 neutralization, one batch of protein was used and three or four different batches of pseudotyped S VSV were used.

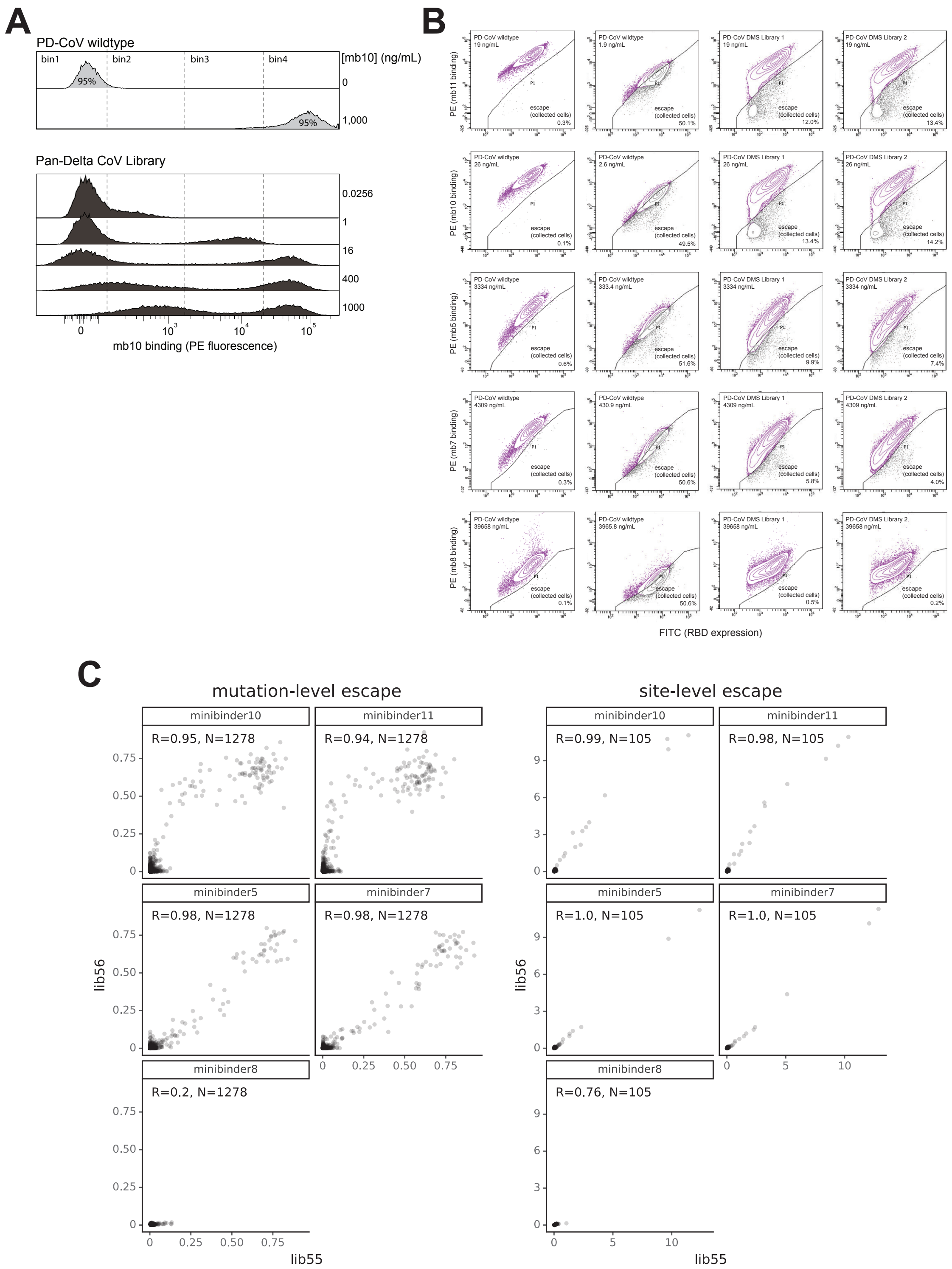

Fig. S5., related to Figure 2: Supporting material for FACS-seq yeast-display experiments. A, FACS gating scheme for evaluation of binding to the DCoV RBD library (MB10 shown for representative data). Yeast-displayed RBDs were incubated with five concentrations of minibinders and sorted into bins from low to high binding followed by deep sequencing. **B**, FACS gating scheme for PDCoV DMS escape mapping. Yeast-displayed RBD mutants were incubated

at the EC_{90} minibinder concentration, and cells escaping binding by ~10x or more were sorted for sequencing to identify binder-escape variants. **C**, Correlation plots between independently sorted DMS libraries for the effects of single amino acid mutations (left) or the sum of all mutant escapes per site (right).

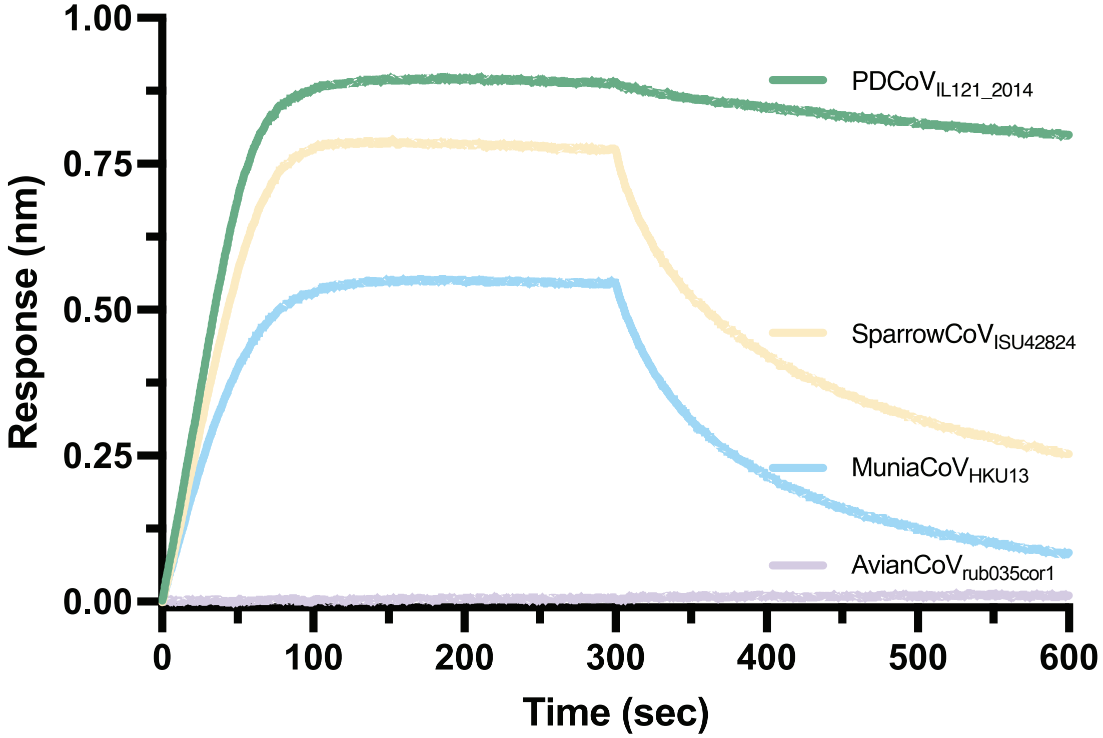

Fig. S6., related to Figure 2: Biolayer interferometry analysis of binding of DCoV RBDs to MB11 (biological replicate 2). A, BLI analysis of PDCoV$_{IL121_2014}$, SparrowCoV$_{ISU42824}$, MuniaCoV$_{HKU13}$, and AvianCoV$_{rub035cor1}$ RBDs at a concentration of 50 nM binding to MB11 immobilized on streptavidin biosensors. Measurements were performed with two distinct batches of MB11 and RBDs.

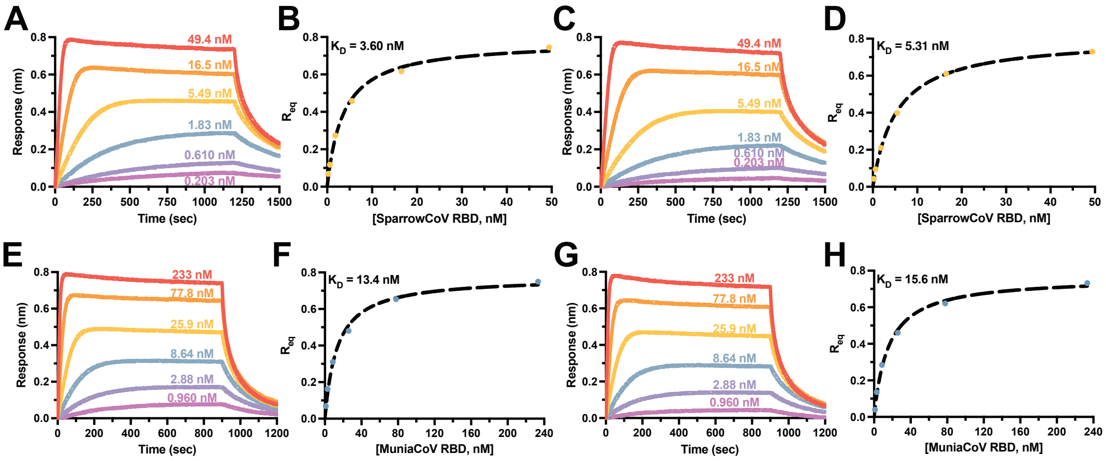

Fig. S7., related to Figure 2: Biolayer interferometry analysis of binding of DCoV RBDs to MB11. A,C, BLI analysis of binding kinetics of the SparrowCoV$_{ISU42824}$ RBD at multiple concentrations to MB11 immobilized at the surface of streptavidin biosensors. The distinct SparrowCoV$_{ISU42824}$ RBD concentrations are indicated according to the color key. **B,D,** The fit to the data of R_{eq} versus SparrowCoV$_{ISU42824}$ RBD concentration using a steady-state model is shown as dashed black lines. Panels A,C and panels B,D correspond to two biological replicates using independently produced batches of proteins. **E,G,** BLI analysis of binding kinetics of the MuniaCoV$_{HKU13}$ RBD at multiple concentrations to MB11 immobilized at the surface of streptavidin biosensors. The distinct MuniaCoV$_{HKU13}$ RBD concentrations are indicated according to the color key. **F,H,** The fit to the data of R_{eq} versus MuniaCoV$_{HKU13}$ RBD concentration using a steady state model is shown as dashed black lines. Panels E,G and panels F,H correspond to two biological replicates using independently produced batches of proteins K_D: equilibrium dissociation constant.

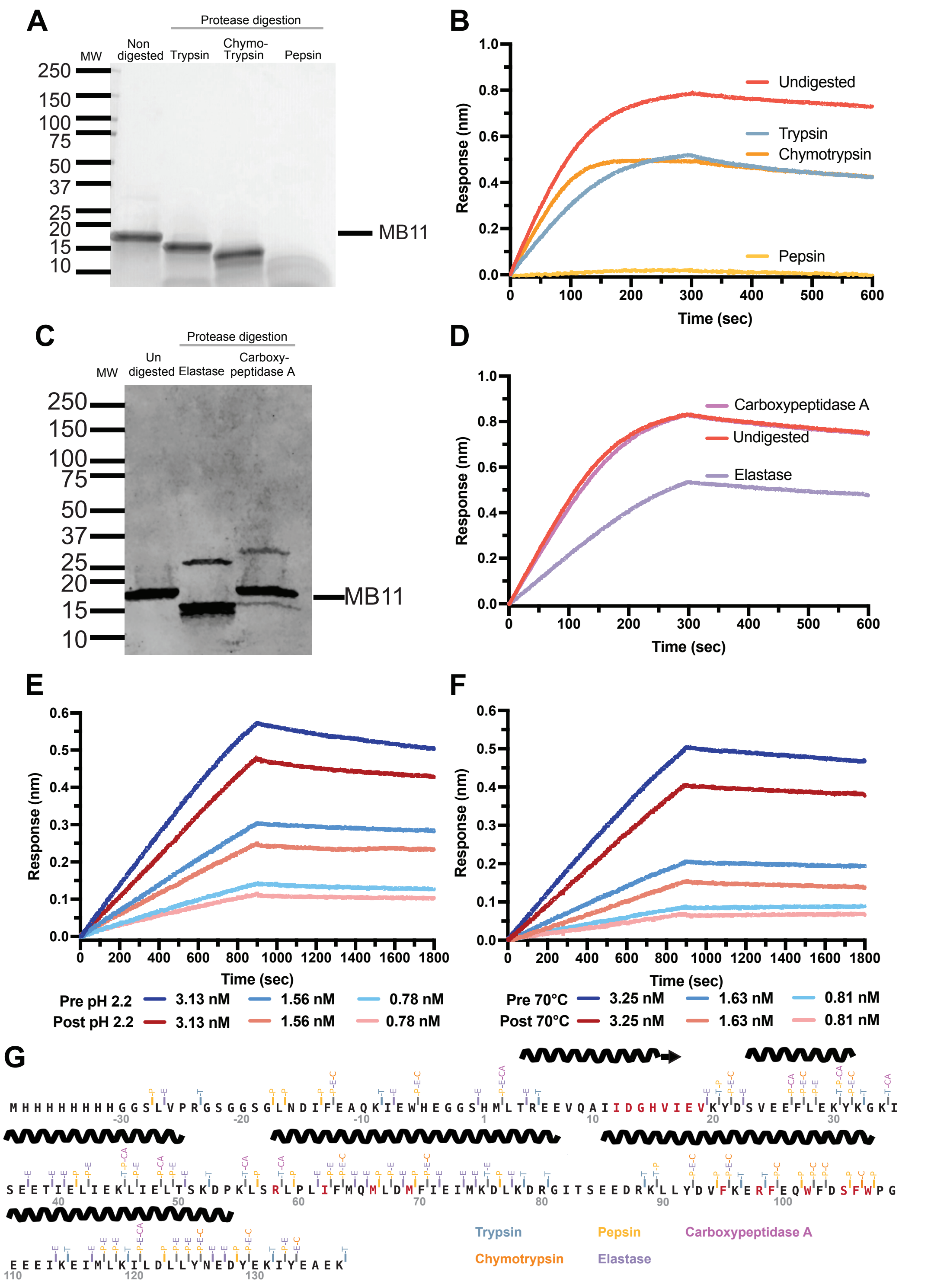

Fig. S8., related to Figure 3: SDS-PAGE of MB11 and biolayer interferometry analysis of binding of the PDCoV$_{IL121_2014}$ RBD to MB11 before and after protease digestion, incubation at pH 2.2 or 70°C (biological replicate 2). A, SDS-PAGE analysis of 5 μg MB11 undigested or digested with 1 μg trypsin, chymotrypsin, or pepsin for 2 hours at 37°C. **B,** BLI analysis of MB11 binding at a concentration of 25 nM to the PDCoV$_{IL121_2014}$ RBD immobilized to streptavidin biosensors, before and after protease treatment as in A. **C,** SDS-PAGE analysis of 5 μg MB11

undigested or digested with 1 µg elastase or carboxypeptidase A for 2 hours at 37°C. **D,** BLI analysis of MB11 binding at a concentration of 25 nM to the $PDCoV_{IL121\_2014}$ RBD immobilized to streptavidin biosensors, before and after protease treatment as in C. **E,** BLI analysis of $PDCoV_{IL121\_2014}$ RBD binding to MB11 immobilized to streptavidin biosensors pre and post MB11 incubation at pH 2.2 for 2 hours. **F,** BLI analysis of $PDCoV_{IL121\_2014}$ RBD binding to MB11 immobilized to streptavidin biosensors pre and post MB11 incubation at 70°C for 1 hour. All BLI measurements were performed with two independent batches of MB11 and RBD. **G,** Prediction of protease cleavage sites in the MB11 sequence using PeptideCutter (1) (trypsin, chymotrypsin, and pepsin) and ProsperousPlus (2) (elastase and carboxypeptidase A). We note that carboxypeptidase A is an exoprotease. Secondary structural elements are annotated based on our structural data. PDCoV-interacting residues (≤ 4 Å) are colored red.

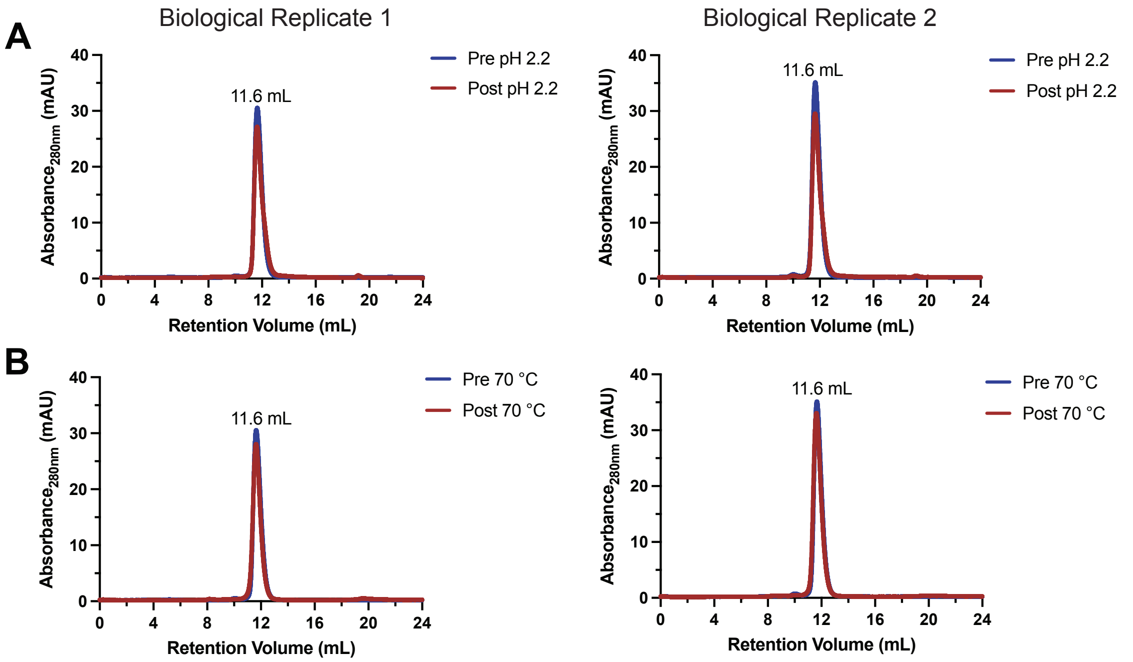

Fig. S9., related to Figure 3: Size exclusion chromatography of MB11 before and after incubation at pH 2.2 or at 70°C. 100 µg of MB11 at 0.25 mg/mL was incubated at pH 2.2 for 2 hours or 70°C for 1 hour. **A,** Analytical SEC of 100 µg of MB11 pre and post pH 2.2 incubation. **B,** Analytical SEC of 100 µg of MB11 pre and post 70°C incubation. Measurements were performed with two independent batches of MB11.

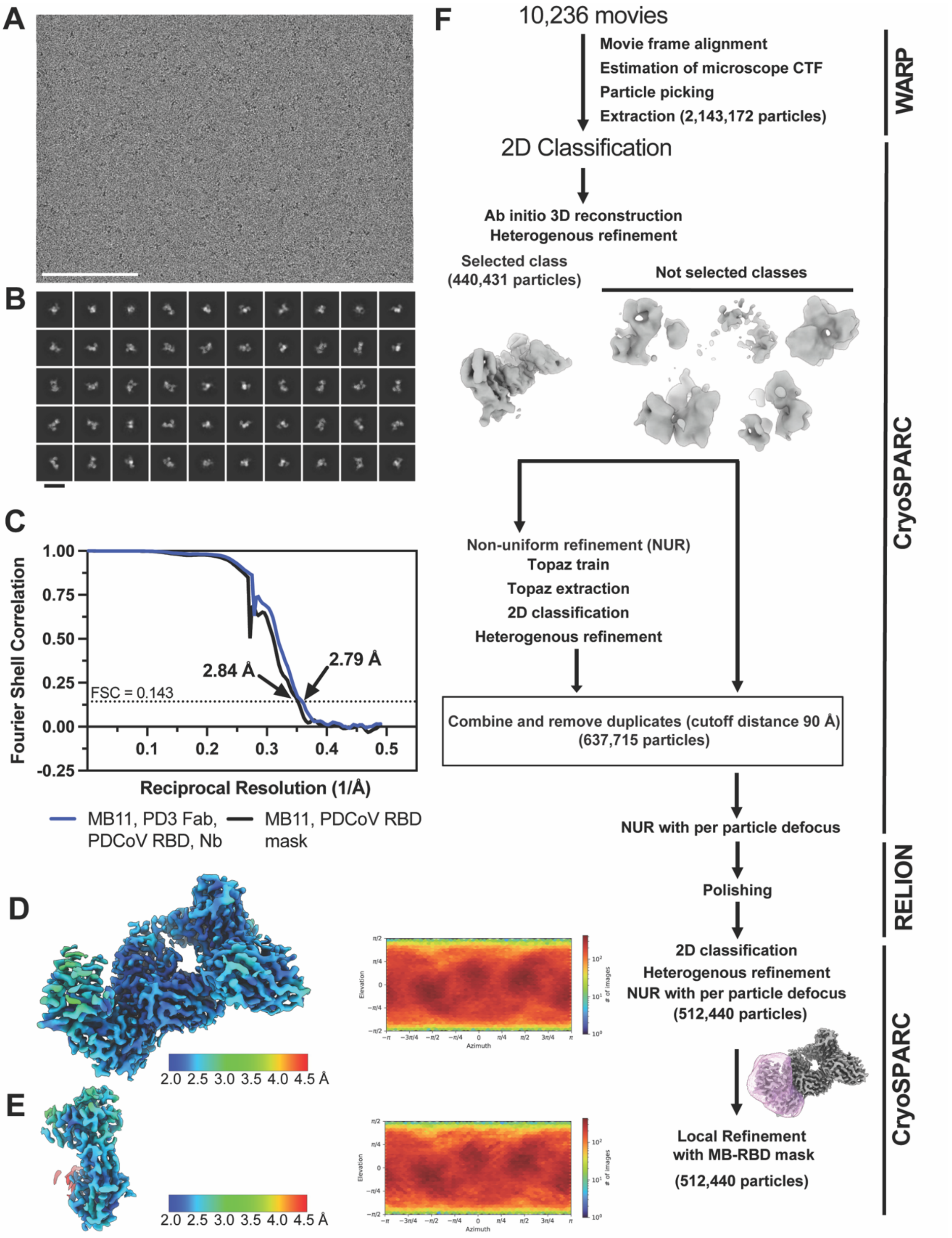

Fig. S10., related to Figure 4: Cryo-EM data collection and processing of the complex between the PDCoV$_{IL121_2014}$ RBD, MB11, PD3 Fab and anti-kappa light chain nanobody. A, Representative electron micrograph (1.64 µm defocus and scale bar = 120 nm). **B,** 2D class averages (scale bar = 120 Å). **C,** Gold-standard Fourier shell correlation curve for the overall refinement (blue) and the local refinement of the PDCoV$_{IL121_2014}$ RBD-MB11 region (black). **D,** 3D reconstruction of PDCoV$_{IL121_2014}$ RBD bound to MB11, PD3 Fab and anti-kappa light chain

nanobody, colored by local resolution calculated with CryoSPARC with the corresponding angular distribution plot shown on the right. **E,** 3D reconstruction of the locally refined region comprising the $PDCoV_{IL121\_2014}$ RBD and MB11 colored by local resolution calculated with CryoSPARC with the corresponding angular distribution plot shown on the right. **F,** Cryo-EM data processing flowchart.

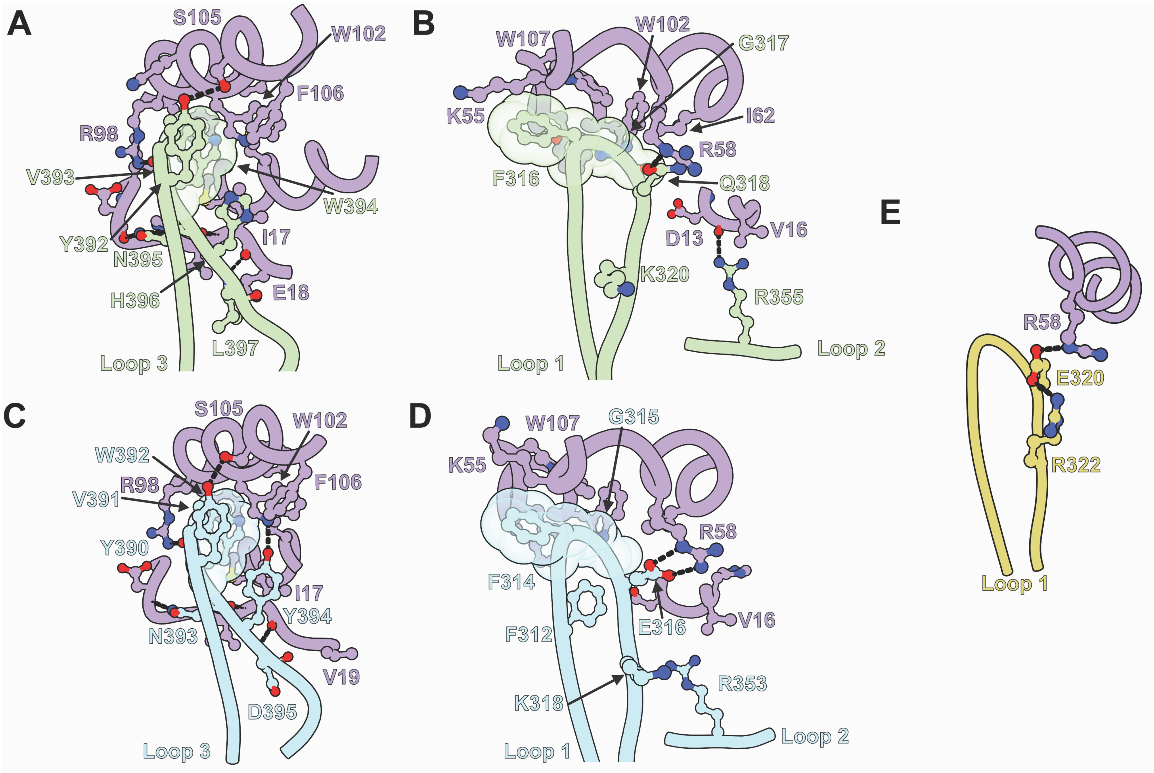

Fig. S11., related to Figure 3: AlphaFold 3-predicted structures of MB11 bound to DCoV RBDs. AF3 was used to predict the structures of MB11 bound to SparrowCoV$_{ISU42824}$ and to MuniaCoV$_{HKU13}$ RBDs. **A-B,** Predicted structure of MB11 (purple)-bound SparrowCoV$_{ISU42824}$ RBD (green) emphasizing interactions with loop 3 (A) and loops 1 and 2 (B). **C-D,** Predicted structure of MB11 (purple)-bound MuniaCoV$_{HKU13}$ RBD (light blue) emphasizing interactions with loop 3 (C) and loops 1 and 2 (D). **E,** Cryo-EM structure of MB11 (purple)-bound PDCoV$_{IL121_2014}$ RBD (gold) emphasizing interactions with loop 1.

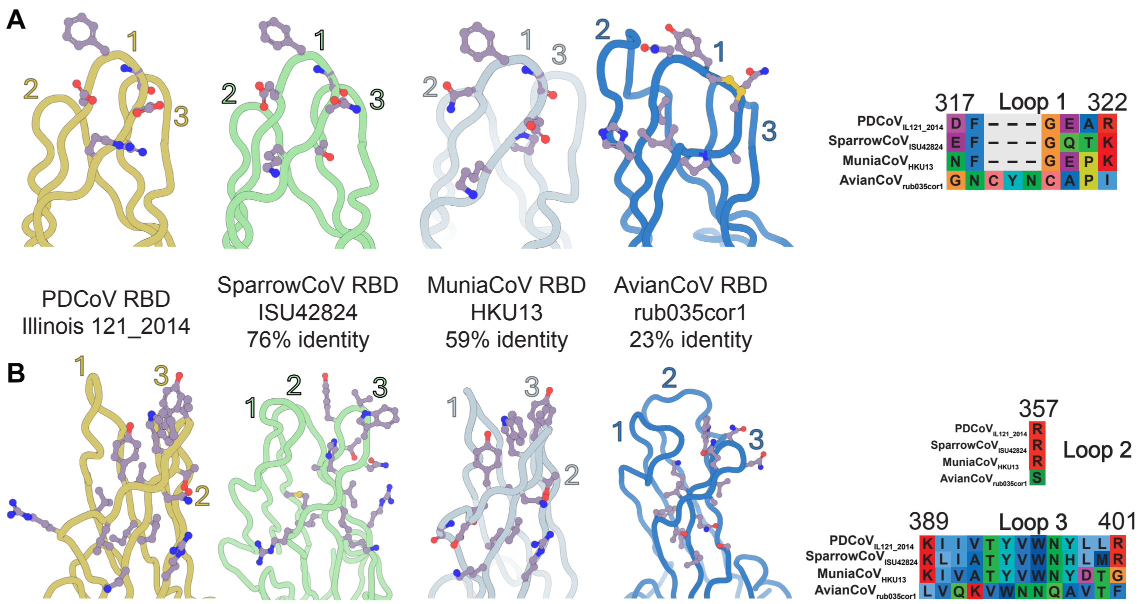

Fig. S12., related to Figure 3: AlphaFold modeling of DCoV RBDs. AF3 was used to predict the $\text{PDCoV}_{\text{IL121\_2014}}$, $\text{SparrowCoV}_{\text{ISU42824}}$, $\text{MuniaCoV}_{\text{HKU13}}$, and $\text{AvianCoV}_{\text{rub035cor1}}$ RBD structures. The structure and sequence of the $\text{PDCoV}_{\text{IL121\_2014}}$ RBD were aligned with that of the other DCoVs using FoldMason (3). **A,** DCoV RBD loop1 predicted structure and sequence. **B,** DCoV loops 2 and 3 predicted structures and sequences. Sequence numbering corresponds to $\text{PDCoV}_{\text{IL121\_2014}}$ S. Select residues are shown for each loop.

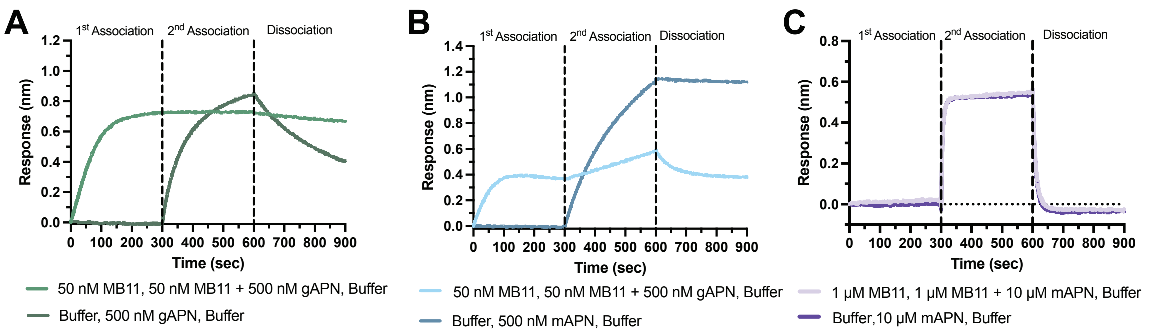

Fig. S13., related to Figure 4: Competition BLI of APN and MB11 binding to DCoV RBDs (biological replicate 2). A, BLI analysis of binding of 500 nM galline APN (gAPN) to the PDCoV$_{IL121_2014}$ RBD immobilized to streptavidin biosensors with and without prior association of MB11 at a concentration of 50 nM. **B,** BLI analysis of binding of 500 nM Munia APN (mAPN) to the MuniaCoV$_{HKU13}$ RBD immobilized to streptavidin biosensors with and without prior association of MB11 at a concentration of 50 nM. **C,** BLI analysis of binding of 10 μM mAPN to the AvianCoV$_{rub035cor1}$ RBD with and without prior association of MB11 at a concentration of 1 μM. BLI analysis was performed in biological duplicate with two independent batches of APN, RBD, and MB11.

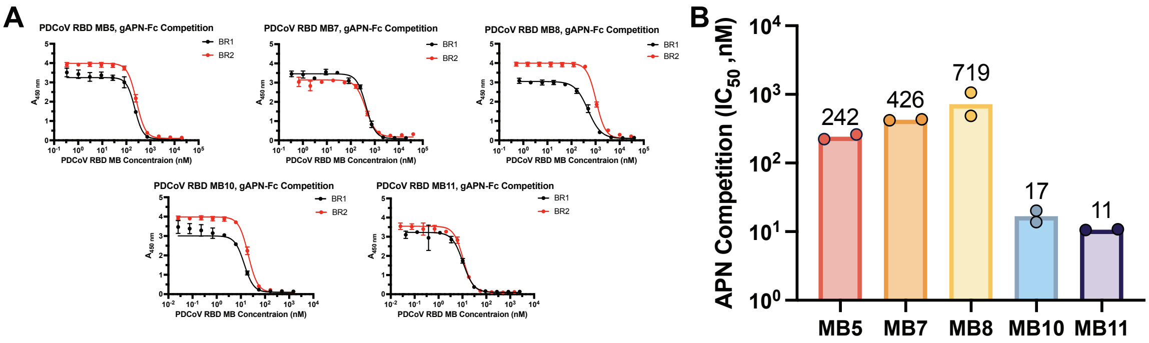

Fig. S14., related to Figure 4: Competition ELISA of gAPN-Fc and MBs for binding to the PDCoV$_{IL121_2014}$ RBD. A, IC_{50} determination of MBs against gAPN-Fc binding. **B,** IC_{50} values from competition ELISAs. 150 µg of PDCoV$_{IL121_2014}$ RBD was immobilized on neutravidin-coated plates and incubated with 15 nM of gAPN-Fc plus 1:3 dilutions of MB starting at 20 µM (MB5), 20 or 40 µM (MB7), 30 or 40 µM (MB8), and 2.5 or 1.25 µM (MB10 and 11). Two biological replicates with two batches of MBs, RBD, and gAPN-Fc were performed.

Tables

Table S1. Sequences of selected PDCoV minibinders.

Design	Sequence
PDCoV$_{IL121_2014}$ RBD Minibinder 1	MTNGWSAHPAEFNEVVLAEERRLLEVVRETGSLEEFERVAAEAAARVEAAWRALGRTPTDDPTDPVWFFRHTLERLRGWTPRERADYFEFLIADITEAL
PDCoV$_{IL121_2014}$ RBD Minibinder 2	TVYMMWRPDPERPGHWVFELYIELPNDPELIEKMKEKAKEVIEEALNDPRYSFIERAEIEERVVKPDVTELIVTFYLDITSEEEKHKQIPVLHEVYDKFKKKVEELLK
PDCoV$_{IL121_2014}$ RBD Minibinder 3	TVYMMWRPDPKRPGYWVFELYIEVPNDEELIKKVKEKAKEVIEEALSDPRFSFIESAEISERKVKDDVTEIIVTFHLDIRSEEEKHKQIPVLHELYDKFKKKLEELLK
PDCoV$_{IL121_2014}$ RBD Minibinder 4	MVMSINEVQLELMEWIDANHPDGDKLISEIHKKQRESLKLWEEGKVEEAKKKMGEIIIETARYLYEVEKVMPEEKVEEIEKKVKEALG
PDCoV$_{IL121_2014}$ RBD Minibinder 5	MDLKLSKEAAKIIAEVLEKNYEKLLRWKHEHFEKLKAENPEKAEEIQVVYDFLAWFHFMMRNHFIDYAEGRMTFEEFLEDLRENVAQNPEVMAKLRSEESLEIYKEYIEKSPLLDVLQEIANKLSPEEVELFNEMLKAVQDIFFPGRPEMHITIEPKP
PDCoV$_{IL121_2014}$ RBD Minibinder 6	MVKSINEVQLELMEYIDANDEDGDKIISEIHERQRESLELWENGKVEEAKKKMAEIIIETAEYLYKEKKVMPKEKVEEIKKKVEEAIG
PDCoV$_{IL121_2014}$ RBD Minibinder 7	SNLELSKEALEIVAEVLEKNYEKLLREKHEYFEKLKKENPEKAEDITVVYDFLAWFHFMLRNHFIDMAEGRMTFQEFLDDLRENVAQNPEVMAKLRSETSLEIYKEYLEKSSLLDVLQEIANRLSPEEQELFNQFLKEVQSIFFPGRPEMDITIEPKP
PDCoV$_{IL121_2014}$ RBD Minibinder 8	SEELFEKLTWLQFYLEQLKAYVEYLKKYKLGVPEEKIEEMEKLLEELEKGLKEVIDALPEEEKKKLEEELERMKEYAKVWNKGFEEEPKPSHHEGEYEVEVYNPWFKTKHTIVFWEESFENQLNHVKELTKWLEENLEKYIEEAKAS
PDCoV$_{IL121_2014}$ RBD Minibinder 9	SGMELWKAQEEMVELMKENPKLKEIHDKIEWEVLEPYWDRPMSDEEYEEYLEKIRQLYIDNGMSEEVVERVMEIYKVHLDYWRSTR
PDCoV$_{IL121_2014}$ RBD Minibinder 10	MKLTEEEVQEIIDGHVIEVKYDSVEEFLEKYKGKISEETIELIKKLIELTSKDPKLSRLPLIFMEMLEMFINIIGDLIEQGITSKENLDLLYEVFKKRFDQWFDSFWPGKDEIKEIMLEILDLIYEKDYKKIYEALK
PDCoV$_{IL121_2014}$ RBD Minibinder 11	HMLTREEVQAIIDGHVIEVKYDSVEEFLEKYKGKISEETIELIEKLIELTSKDPKLSRLPLIFMQMLDMFIEIMKDLKDRGITSEEDRKLLYDVFKERFEQWFDSFWPGEEEIKEIMLKILDLLYNEDYEKIYEAEK
PDCoV$_{IL121_2014}$ RBD Minibinder 20	MVKIYIMKDGEEKEIELPIEWDRENPQEVHKKVMEYFEKYEPGLKKNEIYYVVD

PDCoV$_{IL121_2014}$ RBD Minibinder 21	TVVEKMKAHVLKDYEYVKKNRPEWVEEITPLVDRIVEAAEAGAS<br>EEEVIRLTGEYWGYMMYKRDIEGKKRKIDYQIIPPEGVSKEVVIEF<br>LVETFKSLVDLYFKDLSPEKALEYAARELLITAVQIHNIELGEFDYE<br>LQDEVEKIIDAPPEERIEFVRKTYPDNEYIQELVDYVLNN
PDCoV$_{IL121_2014}$ RBD Minibinder 29	SEELFKKLTWYHFYYEQLKAYVEYLKKYKLGVPQEEIDEMEEKLE<br>EYKKILEEVKKALPEEKQKELEEELKRMSEYAEEWKKGFEEEPK<br>PSHHPGEYEVEVYNPWFGTKHTIVFWPESFENQKKHIEKLTEEL<br>KKNKEELIKEAKEI
PDCoV$_{IL121_2014}$ RBD Minibinder 50	MVREKMKAHVLKDWEYVKEHRPEWVEEIEPLVKKIVEAVEKGAS<br>DEEVIKLTGEYWGYMMFKRDSEGKKRKIDYQIIPPEGVSPEVVVD<br>FYVATFKSLVDKYFEDYDKEKALEYSSRELLIYSVQIHNIEKGEFD<br>YELQDKVEELIDAPPEERIEFVRETYPDNEAVNELVDYILNN
PDCoV$_{IL121_2014}$ RBD Minibinder 52	PTPWNSYETPFTVMFWFLLYQVAESVRDMKEKEDGEKIAVQRIS<br>RLPESLKNMYRSQKELYEEVKPKEKWSEEEIPAVELAEKLVEMID<br>TGASEEERRELAKEAVKLLKVESKYSPFAKLVLEVFSDEELEELL<br>EKGLELIQLVREDKAATGKVDEKRVVEKIKEIGEKFGEKLEKFLEA<br>FEKLRKEMGV
PDCoV$_{IL121_2014}$ RBD Minibinder 95	MKMTKEEIIKRLAELAEKNDNSIIEIEGTMYWQIPARNRVLQSDFW<br>MRINPEDKDNVENVESWMYMEVLPSEYLDLNAEDKIEVKIEGIET<br>DPETGKKVIKVSFKAYFRAETEQEVWDKRIEVFEEIIKKLESL

Table S2., related to Figure 1: Kinetics and affinity of PDCoV$_{IL121_2014}$ RBD binding to MB11. Two batches of each protein were used.

Biological Replicate	K_D (M)	K_a (1/Ms)	K_d (1/s)
1	1.75×10^{-10}	1.55×10^{5}	2.72×10^{-5}
2	1.35×10^{-10}	5.84×10^{4}	7.88×10^{-6}

Table S3., related to Figure 4. Cryo-EM data collection and refinement statistics.

	$PDCoV_{IL121\_2014}$ RBD with MB11, PD3 Fab, and anti-kappa light chain nanobody<br><br>PDB: 11ZW<br>EMD-76233	$PDCoV_{IL121\_2014}$ RBD with MB11, PD3 Fab, and anti-kappa light chain nanobody (local refinement of the region comprising MB11 and the RBD)<br><br>PDB: 11ZV<br>EMD-76232
**Data collection and processing**		
Magnification	105,000	105,000
Voltage (kV)	300	300
Electron exposure ($e^-/Å^2$)	52.8	52.8
Defocus range (μm)	-0.5 - -1.5	-0.5 - -1.5
Pixel size (Å)	0.829	0.829
Symmetry imposed	C1	C1
Final particle images (no.)	512,440	512,440
Map resolution (Å)	2.8	2.8
FSC threshold	0.143	0.143
Map sharpening B factor ($Å^2$)	-116.8	-117
**Validation**		
MolProbity score	0.80	0.60
Clashscore	0.61	0.26
Poor rotamers (%)	0.87	0.52

Bonds (RMSD)		
Length (Å) (# > 4σ)	0.007 (0)	0.004 (0)
Angles (°) (# > 4σ)	1.318 (12)	0.760 (1)
Ramachandran plot		
Favored (%)	97.51	98.31
Allowed (%)	2.49	1.69
Disallowed (%)	0.00	0.00

Table S4., List of intermolecular contacts between PDCoV$_{IL121_2014}$ RBD and MB11 (≤ 4Å) and human APN (4).

**PDCoV$_{IL121_2014}$ RBD Residue**	**MB11 Residue**	**hAPN Residue**
M316	F106	-
F318	I62, F106, W107	D315, Y316, N319, F369, P371, K379
G319	I62, W102, F106	P371
E320	R58, I62	K379, E426, W429
R322	R58	K379, E426, T428
R357	D13, G14, V16	N736, N738, E742
Y394	S105, F106	L372
V395	R98, W102	L372
W396	I12, I17, M66, M69, F95, R98, F99, W102	L372, N786, P787, I788, H789, P790, R793
N397	D13, H15, V16, I17	R741, E742, I743
Y398	I17, W102	I743, H789
L399	I17, E18	N736, E742, I743, P744, E745
R401	V19	E745, N746, D749

Table S5. DNA constructs used in this study.

Construct	GenBank ID	Residues	Vector	Signal Peptide	Cleavage Site	Tags	Tag Location
PDCoV$_{IL121_2014}$ S1-Fc	KJ481931.1	1-545	pcDNA3.1+	Native	Thrombin	Human Fc	C-terminus
PDCoV$_{IL121_2014}$ S RBD	KJ481931.1	303-415	pcDNA3.1+	µ-phosphatase	Thrombin	AVI and 8x HIS	C-terminus
SparrowCoV$_{ISU42824}$ S RBD	MG812377.1	301-420	pcDNA3.1+	µ-phosphatase	Thrombin	AVI and 8x HIS	C-terminus
MuniaCoV$_{HKU13}$ S RBD	FJ376622	299-418	pcDNA3.1+	µ-phosphatase	Thrombin	AVI and 8x HIS	C-terminus
AvianCoV$_{rub035cor1}$ S RBD	MT138108	327-459	pcDNA3.1+	µ-phosphatase	Thrombin	AVI and 8x HIS	C-terminus
Galline APN-Fc	ACZ95799.1	66-967	pcDNA3.1+	µ-phosphatase	Thrombin	Human Fc	C-terminus
Munia APN-Fc	XP_021397454.1	117-1012	pcDNA3.1+	µ-phosphatase	Thrombin	Human Fc	C-terminus
PD3 Fab Heavy Chain	N/A	1-223	pcDNA3.1+	CD5	None	8x HIS	C-terminus
PD3 Fab Light Chain	N/A	1-214	pcDNA3.1+	CD5	None	None	N/A
PDCoV$_{IL121_2014}$ S FL	KJ481931.1	1-1139	pcDNA3.1+	Native	None	FLAG	C-terminus
SparrowCoV$_{ISU42824}$ S FL	MG812377.1	1-1136	pcDNA3.1+	Native	None	FLAG	C-terminus

MuniaCoV$_{HKU13}$ S FL	FJ376622	1-1135	pcDNA3.1+	Native	None	FLAG	C-terminus
AvianCoV$_{rub035cor1}$ S FL	MT138108	1-1179	pcDNA3.1+	Native	None	FLAG	C-terminus
Galline APN FL	ACZ95799.1	1-967	pcDNA3.1+	Native	None	FLAG	C-terminus
Sparrow APN FL	XP_039579472.1	1-1214	pcDNA3.1+	Native	None	FLAG	C-terminus
Munia APN FL	XP_021397454.1	1-1119	pcDNA3.1+	Native	None	FLAG	C-terminus

μ-phosphotase signal peptide: MGILPSPGMPALLSLVSLLSVLLMGCVAETGT
CD5 signal peptide: MPMGSLQPLATLYLLGMLVASVLA

SI References

1. E. Gasteiger, *et al.*, “Protein identification and analysis tools on the ExPASy server” in *The Proteomics Protocols Handbook*, (Humana Press, 2005), pp. 571–607.

2. F. Li, *et al.*, ProsperousPlus: a one-stop and comprehensive platform for accurate protease-specific substrate cleavage prediction and machine-learning model construction. *Brief. Bioinform.* **24** (2023).

3. C. L. M. Gilchrist, M. Mirdita, M. Steinegger, Multiple protein structure alignment at scale with FoldMason. *Science* **391**, 485–488 (2026).

4. W. Ji, *et al.*, Structures of a deltacoronavirus spike protein bound to porcine and human receptors. *Nat. Commun.* **13**, 1467 (2022).
